# Strategies for Designing Circular, Sustainable, and Nonpersistent Consumer Plastic Products: A Case Study of Drinking Straws

**DOI:** 10.1021/acs.est.5c05448

**Published:** 2025-08-22

**Authors:** Bryan D. James, Yanchen Sun, Kali Pate, Brenden Irving, Collin P. Ward

**Affiliations:** † Department of Marine Chemistry and Geochemistry, 10627 Woods Hole Oceanographic Institution (WHOI), Woods Hole, Massachusetts 02543, United States; ‡ Department of Chemical Engineering, 1848 Northeastern University, Boston, Massachusetts 02115, United States

**Keywords:** ecodesign, design for degradation, design for environment, sustainable engineering, plastic pollution, biodegradation

## Abstract

Drinking straws present a simple form factor for evaluating material design strategies taken by manufacturers to achieve circular, sustainable, and nonpersistent products in response to global restrictions and consumer sentiment. We investigated 13 on-the-market drinking straws of varying formulations, characterizing their physical, chemical, and marine biodegradation properties. These data informed sustainability metrics used for evaluating the effectiveness and trade-offs of design strategies, ultimately arriving at four key conclusions. First, diverting anthropogenic methane as an alternative feedstock to make polyhydroxyalkanoates (PHAs) resulted in the only straw with a net-negative global warming potential. Second, adding biogenic fillers to conventional polymers (e.g., polypropylene) to minimize plastic usage is unlikely to produce substantial environmental benefits compared to using alternative polymers. Third, many marketing claims about circularity, sustainability, and persistence were unsupported, likely magnifying the mismanagement and environmental impacts of these products. Fourth, improper disposal of compostable straws in landfills could increase the global warming potential of the item by up to 6 times and offset numerous advantages afforded by biodegradable materials, thereby warranting greater investment in waste management infrastructure. The analysis of design strategies and their trade-offs provided herein should be applied broadly when developing and adopting future consumer products.

## Graphical abstract

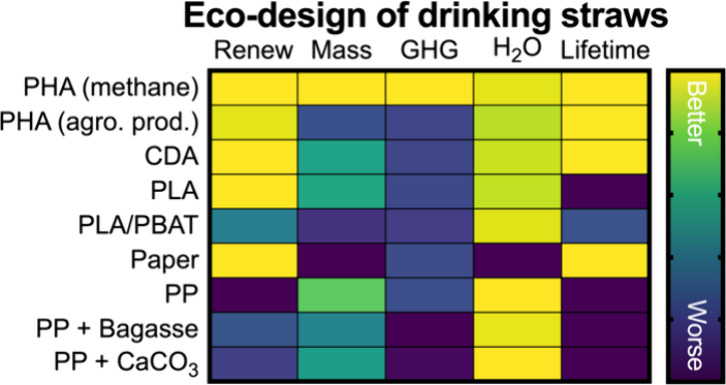

## Introduction

In the face of the plastic pollution crisis, manufacturers of plastic products are challenged to produce functional and profitable products while at the same time meet the demands of policymakers and the public to use more sustainable materials and processes. Presently, there is no single, definite answer to this complex engineering design problem, which is exacerbated by the lack of a uniform or agreed upon definition for sustainability across sectors (i.e., government, industry, the public, etc.). For example, should a product be made from biobased, but persistent polypropylene (PP) or fossil-based, but compostable poly­(butylene adipate terephthalate) (PBAT)? Making the most appropriate material selection decision is imperative because, once entrenched, transitioning products to new materials faces significant inertia. It is currently unclear what successes and pitfalls companies are facing while addressing this multiobjective design problem of functionality, profitability, circularity, sustainability, and persistence. An analysis of the product landscape and design strategies of drinking straws may provide clarity surrounding this design problem.

Drinking straws are polarizing, frequently littered, and pervasive single-use products. Upward of 50 billion drinking straws are used annually in the U.S.,^1^ and these products are often improperly disposed of (mismanaged), constituting ∼5% of the plastic debris collected from coastlines globally.^2^ To curb their deleterious impacts as pollution, regulations have targeted drinking straws globally from the municipal to federal levels, banning products perceived as persistent in the environment. In response to these restrictions and consumer sentiment, a market for more sustainable and less persistent drinking straws emerged. In recent years, the market has ballooned to include dozens of brands (Table S1) claiming to be less impactful to the environment. Such claims are predicated on the switch to plastic formulations using different polymers, such as polyhydroxyalkanoates (PHAs), and additives, such as organic and inorganic fillers.

Environmentally conscious consumers, businesses, and policymakers face challenges navigating advertising for these new drinking straws.^3^ The details provided by brands and manufacturers range from cryptic and vacuous marketing language to informative and readily accessible messaging. This fact is belabored by the myriad certifications and labels on product packaging that require a detailed understanding of their test methods to fully appreciate the nuances and reasonable claims that can be drawn from them. Two of the most frequently used labels are from certifiers TÜV Austria and the Biodegradable Products Institute (BPI),^4,5^ which provide certificates for home and industrial compostability. These third parties certify polymer resins and products according to compostability standards (e.g., ASTM 6400 ^6^), as well as their additional scrutiny. Other labels reflect claims of feedstock renewability and global warming potential (GWP). The emphasis on certification is a legal matter (e.g., under the U.S. Federal Trade Commission Act^7^). Certification is necessary for businesses to substantiate any environmental marketing claims, particularly those related to degradability.

Addressing persistence has often been met by designing straws for degradation in engineered environments, such as industrial composting facilities. This attribute is necessary for circular material schemes following a managed life cycle. Yet, the safeguard solution to the pollution of frequently mismanaged items is to imbue the items with inherent degradability in all environments: engineered and natural.^8^ This feature has been advocated as one of six key interventions for mitigating the potential impacts of plastic pollution by embracing the principles of green chemistry and engineering in material and product design.^9–11^

While many drinking straws are certified as compostable and claim biodegradability, few present evidence for their degradation in the marine environment, where these items are ubiquitous.^12^ Many compostable polymers feature incongruent degradation behavior with natural environments [e.g., poly­(lactic acid) (PLA)].^13–15^ Additionally, while certificates for marine biodegradability exist, the methods for evaluating marine degradation do not represent natural conditions because of well-recognized bottle effects.^13^ In short, these methods provide evidence that marine microbes can biodegrade a material, but they are incapable of estimating lifetimes in natural settings. Having intrinsic biodegradability in the marine environment is necessary for plastic products, such as drinking straws, that frequently leak into the ocean.^8^

Plastic pollution is not the only environmental impact drinking straw manufacturers have aimed to avoid and reduce by switching to alternative plastic formulations. Impacts across the entire life cycle are being addressed to varying extents. The approaches manufacturers take to reduce the life cycle environmental impacts of drinking straws represent a microcosm of the broader landscape of approaches tried by the plastics industry to address the impacts of feedstock and end-of-life (EoL) disposal. Thus, drinking straws present a simple form factor (a hollow cylinder) invariant across brands for quantifying the environmental impacts of different commercially viable material design strategies applied at scale for achieving sustainable plastic products.

Herein, drinking straws of varying formulations from different manufacturers were investigated for their environmental impacts. These straws encompass products manufactured by startups to multinational corporations being adopted by local stores and international brands alike. The straws were evaluated for their physical, chemical, and realistic marine biodegradation properties (i.e., lifetimes and microbial community composition), which informed, as part of an eco-audit,^16^ sustainability metrics related to renewability, material efficiency, resource utilization, GWP, and environmental persistence. This analysis identified successful design strategies for sustainability and circularity, as well as their trade-offs that should be applied broadly in designing future plastic products.

## Materials and Methods

### Materials

The drinking straws experimentally evaluated in this study included three PHA-based straws produced by Aircarbon, LifeMade, and Pura Vida Bioplastic, one calcium carbonate-filled straw made with an undisclosed resin produced by Strawfish, one agave bagasse-filled straw made with an undisclosed resin and produced by the Sustainable Agave Company, and two straws made from proprietary or undisclosed resins produced by Matter and LOLIWARE. An uncoated paper straw and a conventional PP straw were used as representative biodegrading (positive control) and nonbiodegrading (negative control) straws, respectively. The Aircarbon, LifeMade, and Pura Vida Bioplastic straws were labeled PHA1, PHA2, and PHA3. The Strawfish and Sustainable Agave Company straws were labeled “CaCO_3_ filled Resin” and “Agave Bagasse filled Resin”. The Matter and LOLIWARE straws were labeled Resin 1 and Resin 2. The positive and negative control straws were labeled Paper and PP. The straws were characterized to have sufficient information for comparison and calculating sustainability metrics (see Extended Materials and Methods, Material characterization, in the Supporting Information). Details (Section S1) and findings (Table S2) on four additional drinking straws previously investigated by our group were included in the analyses for broader coverage of the design space.^14^ These straws include a cellulose diacetate (CDA) straw provided by Eastman, two PHA straws produced by Phade and Beyond Green (labeled PHA4 and PHA5, respectively), and a PLA straw produced by Plasticless (labeled PLA).

### Evaluation of Marine Biodegradation

The biodegradation of drinking straws under coastal marine conditions was assessed by three orthogonal and previously reported methods: short-term seawater incubations measuring microbial respiration,^17,18^ long-term incubations in a continuous flow natural seawater mesocosm monitoring mass loss,^14,15,17,19–21^ and analysis of microbial community composition.^14,19^ The specific details of each approach are provided in the Extended Materials and Methods in the Supporting Information, Tables S3 and S4 and Figures S1–S6.

### Sustainability Metrics

Several sustainability metrics were calculated, including renewability, material efficiency, embodied greenhouse gas (GHG) emissions, embodied water usage, projected environmental lifetime in the marine environment, and managed and mismanaged EoL GWP. Data were collated from literature sources for material properties not measured or calculated in this study (Table S5). The specific embodied GHG emissions for the biogenic filler materials (CaCO_3_ and agave bagasse) were assumed to be negligible, i.e., they were assumed to be 100% sequestering. Material efficiency is the amount of material necessary to produce a functional product which, in this case, is equivalent to the mass of the straw. Embodied GHG emissions and embodied water usage were calculated as the product of the mass of each straw and the specific embodied GHG emissions and the specific water usage for its polymer and filler (if present), respectively. Renewability was calculated as the fraction of renewable material used in each straw. Managed and mismanaged EoL GWPs were calculated for compost and landfill environments. For calculations, the Agave Bagasse filled Resin was assumed to be 25% agave bagasse (biocomposites of agave-derived bagasse typically include 20–30 wt % bagasse^22–24^ ). Based on the analyses described within, Resin 1 was determined to be a PLA/PBAT blend. It was then assumed that the Resin 1 straw was a commercial blend of PLA and PBAT advertised for profile extrusion of straws, consisting of 70% PLA and 30% PBAT.^25^ PBAT was assumed to be fossil-based.^26^ Metrics were not calculated for the Resin 2 straw because of a lack of reliable data for this material and its exact composition.

#### Embodied GHG Potential of Biogenic Materials

The embodied GHG potential of each biogenic material used in the straws was estimated, assuming all the carbon (C) associated with the material removed atmospheric carbon dioxide (CO_2_). Embodied GHG potential was calculated according to eq 1, where χ_C_ is the weight percent of C in the material (kg C/kg material), $\frac{n_{{\mathrm{CO}}_{2}}}{n_{C}}$ is the stoichiometric ratio of CO_2_ converted to C in the biogenic material (assumed 1), *w*_C_ is the molar mass of C (12 g/mol), and *w*_CO_2__ is the molar mass of CO_2_ (44 g/mol).

1 $$C_{\mathrm{biogenic material}}=\left(\chi_{C}\right)\left(\frac{n_{{\mathrm{CO}}_{2}}}{n_{C}}\right)\left(\frac{w_{{\mathrm{CO}}_{2}}}{w_{C}}\right)$$

The values of χ_C_ were retrieved from reported values in the peer-reviewed literature. Calcium carbonate is 12 wt % C, and agave bagasse has been reported to be 41.25 wt % C.^27^

#### EoL GWP

The EoL GWP of a biodegradable material was estimated for landfill and composting environments. Only contributions from methane (CH_4_) and CO_2_ were included in the estimates. The Buswell equation^28^ was used to estimate the stoichiometric ratios for converting the biodegradable material to CH_4_ and CO_2_. For composting conditions (aerobic conditions), all the carbon in the material was assumed to be converted to CO_2_. The EoL GWP was calculated as the sum of the contributions from CH_4_ and CO_2_ in units of CO_2_-eq/kg material, which was calculated using eq 2, where GWP_CO_2__ is the 100-year global warming potential of CO_2_ equal to 1 kg CO_2_-eq/kg CO_2_, GWP_CH_4__ is the 100-year global warming potential of CH_4_ equal to 28 kg CO_2_-eq/kg CH_4_, $\frac{n_{{\mathrm{CO}}_{2}}}{n_{C}}$ is the stoichiometric ratio of C converted to CO_2_ (assumed 1 for compost and calculated for the material using the Buswell equation for landfill), $\frac{n_{{\mathrm{CH}}_{4}}}{n_{C}}$ is the stoichiometric ratio of C converted to CH_4_ (assumed 0 for compost and calculated for the material using the Buswell equation for landfill), and *w*_CH_4__ is the molar mass of CH_4_ (16 g/mol).

2 $$C_{\mathrm{GWP}}^{\mathrm{EoL}}=\left({\mathrm{GWP}}_{{\mathrm{CO}}_{2}}\right)\left(\chi_{C}\right)\left(\frac{n_{{\mathrm{CO}}_{2}}}{n_{C}}\right)\left(\frac{w_{{\mathrm{CO}}_{2}}}{w_{C}}\right)+\left({\mathrm{GWP}}_{{\mathrm{CH}}_{4}}\right)\left(\chi_{C}\right)\left(\frac{n_{{\mathrm{CH}}_{4}}}{n_{C}}\right)\left(\frac{w_{{\mathrm{CH}}_{4}}}{w_{C}}\right)$$

The calculations for each straw material are provided in the Supporting Information.

### Social Costs

The social costs for the associated GHG emissions due to the straws were calculated according to eq 3,

3 $$V_{\mathrm{GHG}}=\alpha_{\mathrm{GHG}}C_{\mathrm{GHG}}m_{0}$$

where *V*_GHG_ is the social cost of GHG emissions for the straw, α_GHG_ is the exchange constant for GHG emissions, *C*_GHG_ is the specific GHG emissions for the material, and *m*_0_ is the initial mass of the straw. GHG emissions included CO_2_ and CH_4_; a 50% capture efficiency for landfill CH_4_ emissions was assumed.^29^ The social costs related to the marine plastic pollution of the straws were calculated following our previously reported method^30^ and using eq 4,

4 $$V_{P}=\alpha_{P}\chi_{P}f_{P}m_{0}t_{L}\left(\frac{3l_{0}-h_{0}}{6l_{0}}\right)$$

where *V*_P_ is the social cost of marine plastic pollution for the straw, α_P_ is the exchange constant for marine plastic pollution, χ_P_ is the total fraction of plastic leaking into the ocean (0.11), *f*_P_ is the fraction with which drinking straws contribute to the total amount of leaked plastic (0.05), *t*_L_ is the projected lifetime of the straw, *l*_0_ is the initial length of the straw, and *h*_0_ is the initial thickness of the straw.^2,31^ The social cost calculation was adjusted by χ_P_ and *f*_P_ because not every item leaks into the environment.^32^ All dollar values are presented in 2024 U.S. dollars (US$).

## Results

Evaluating the potential environmental impacts of drinking straws requires knowing what material(s) were used to make the straws and the dimensions and properties of the straws. For this case study, nine commercial plastic straws were experimentally investigated: three PHA straws advertised to be from different PHA polymers, four straws of undisclosed resin several of which were advertised to include various fillers, and two conventional straws (PP and paper). These straws were investigated because they represent a mixture of the straws in the marketplace (Table S1) and have received notable press and recognition.^33–35^ Information on the straws was collected by reviewing product marketing, regulatory, and third-party certification documents and databases, as well as characterizing the thickness, mass, density, and composition of the straws. Overall, the available information about the straws varied considerably between brands. Paper straws were generally promoted as biodegradable and compostable, often without emphasis on certifications. For PP straws, marketing materials made little to no mention of sustainability. Despite their differences, the unit price of each straw in 2023 U.S. dollars, when purchased in bulk, ranged from 0.02 to 0.08 US$/straw. The properties of each straw are summarized in Table 1.

**Table 1:** Drinking Straw Properties

straw	unit price [US$/straw]^a^	polymer	filler	mass [mg]^b^	thickness [mm]^c^	density [g/cm^3^]^c^	inorganic content [wt%]^d^
paper	0.03	paper	none reported	1133.0 ± 8.2	0.33 ± 0.02	0.75 ± 0.02	ND
PHA1	0.05	P3HB	none reported	437.8 ± 13.4	0.12 ± 0.01	1.37 ± 0.03	ND
PHA2	0.08	PHA copolymer	CaCO_3_	953.2 ± 29.2	0.19 ± 0.01	1.41 ± 0.03	15.8 ± 1.3
PHA3	0.03	PHBH	none reported	863.1 ± 32.2	0.25 ± 0.01	1.13 ± 0.05	ND
Resin 1	0.05	PLA/PBAT blend	none reported	1028.0 ± 11.0	0.23 ± 0.01	1.22 ± 0.02	ND
Resin 2	0.08	seaweed-based biopolymer blend	CaCO_3_	2117.0 ± 90.4	0.49 ± 0.05	1.30 ± 0.14	30.8 ± 0.1
CaCO_3_ filled Resin	0.02	PP	CaCO_3_	745.7 ± 12.3	0.25 ± 0.02	1.03 ± 0.01	17.8 ± 0.1
Agave Bagasse filled Resin	0.04	PP	Agave bagasse	816.9 ± 10.1	0.29 ± 0.04	0.85 ± 0.01	ND
PP	0.03	PP	none reported	614.5 ± 1.7	0.15 ± 0.01	0.91 ± 0.02	ND

a In 2023 US$ for bulk purchasing.

b Presented as the mean ± standard deviation of five measurements for the product.

c Presented as the mean ± standard deviation of 7–11 measurements.

d Presented as the mean ± standard deviation of three measurements; ND = not detected.

### PHA1

According to the manufacturer’s Web site, product packaging, and regulatory documents,^36,37^ the PHA1 straw was made of a poly­(3-hydroxybutyrate) (P3HB) produced by Newlight Technologies and met several third-party biodegradation certification standards for compostability (TÜV Austria Home and Industrial S2100; BPI Certified).^38,39^ To meet the standards, the certifying bodies prescribed a maximum nominal thickness for the straws of 152 μm (TÜV) and 182 μm (TÜV, BPI), depending on the material and standard. Additionally, promotional materials from the manufacturer labeled the straws as carbon-negative, sequestering 52.5 g CO_2_-eq/straw (119.9 kg CO_2_-eq/kg material) as verified by SCS Global Services following ISO 14044:2006 for manufacturing and disposal.^40^ Carbon sequestration resulted from the use of diverted anthropogenic CH_4_ emissions as its feedstock.^41^ Analysis of the stable and radiocarbon isotopic composition for the straw confirmed a fossil-derived methanogenic C source (Δ^14^C = −1000 ± 0.2‰, δ^13^C = −42.3 ± 0.1‰, and *n* = 3). No inorganic additives were detected (Table 1).

### PHA2

The packaging of the PHA2 straw stated it was made from a PHA material with the trade name Nodax, produced by Danimer Scientific. In March 2025, Danimer Scientific filed for bankruptcy.^42^ Regulatory documents disclosed that the food-contact approved grade of these materials is a random copolymer in which 3-hydroxybutyrate units represent approximately 75% of the polymer, with 3-hydroxyvalerate, 3-hydroxyhexanoate, 3-hydroxyoctanoate, and/or 3-hydroxydecanoate monomer units representing up to 25% of the polymer.^43^ Nodax PHAs are certified biobased (TÜV Austria S0292), using vegetable oils as renewable feedstocks.^39,44^ The straws met several third-party biodegradation standards for compostability (TÜV Austria Home S2594; BPI Certified).^38,39^ To meet the standards, the certifying bodies prescribed a maximum nominal thickness for the straws of 190 μm (TÜV) and 203.2 μm (BPI). While not advertised, it had a measurable inorganic additive content of 15.8 ± 1.3 wt % (*n* = 3). Infrared (IR) spectroscopy and X-ray fluorescence (XRF) were used to determine the composition of the inorganic additive content of the straw. The IR spectrum reflected that of a PHA and included characteristic peaks for CaCO_3_ and elemental analysis by XRF of the inorganic material detected only Ca in significant quantities (47.56% ± 3.16%; *n* = 3), indicating that its inorganic additive content was most likely CaCO_3_ (Figure S7). Calcium carbonate is often used as a filler in plastics to reduce cost and modify mechanical and processing properties of the material. Of TÜV Austria certified home compostable drinking straws, Nodax was one of the most common materials used by different brands (e.g., LifeMade S2594, Biolo S2166, Phade S1328, Eagle Beverage S2332, Urthpact S0589, Greenprint S2380).^39^

### PHA3

According to promotional documents and social media posts,^45,46^ the PHA3 straw was made of poly­(3-hydroxybutyrate-*co*-3-hexanoate) (PHBH) produced by Kaneka Green Planet.^47^ Regulatory documents disclosed that the food-contact grade of the material is poly­(3-hydroxybutyrate-*co*-3-hydroxyhexanoate).^48^ The straws were labeled a U.S. Department of Agriculture Certified 100% biobased product and certified biobased (TÜV Austria S0318), using vegetable oils as renewable feedstocks.^45^ No inorganic additive content was detected (Table 1). The straws met several third-party biodegradation standards for compostability (TÜV Austria Home and Industrial S2541; BPI Certified).^38,39^ To meet the standards, the certifying bodies prescribed a maximum nominal thickness for the straws of 185 μm (BPI) and 320 μm (TÜV). The measured thickness was greater than the specified limit for BPI certification (Table 1).^45^

### Filled Resins

According to the manufacturer’s Web site and promotional materials,^49^ the CaCO_3_ filled Resin straws were made with a resin filled with calcium carbonate and unspecified enzymatic degradation additives.^50^ The calcium carbonate was derived from waste oyster shells sourced from Mexico. Marketing materials claimed the straws could biodegrade in 12–18 months in natural landfills (ASTM D5526 ^51^) and outdoor conditions through aerobic and anaerobic processes.^50^ According to the manufacturer’s Web site,^52^ the Agave Bagasse filled Resin straws were made with material from the agave plant unused in tequila production and were biodegradable.^53^ The formulated resin was claimed to have a lifetime of 10–24 months in soil.^54^ The manufacturers of the CaCO_3_ filled and Agave Bagasse filled Resin straws did not disclose the polymer used to make their straws in marketing materials. The IR spectra for the CaCO_3_ filled Resin and the Agave Bagasse filled Resin straws matched that of PP (Figures S8 and S9). Along with the characteristic peaks for PP, the IR spectrum of the CaCO_3_ filled Resin straw included well-defined peaks for CaCO_3_ at 1450, 1420, and 874 cm^–1^. The measured inorganic additive content was 17.8 ± 0.1 wt % (*n* = 3), presumably all CaCO_3_.

### Resin 1

Product packaging for the Resin 1 straw indicated it was made from NatureStar resin, advertised as a biodegradable and compostable biopolymer.^55,56^ The product Web site stated that, “straws will breakdown within 12 months, leaving only biomass behind which can be used to enhance soil”.^57^ The straw met several third-party biodegradation standards for compostability (TÜV Austria Home S2699; BPI Certified).^38,39^ In conflict with the certifications, the product page on the retailer’s Web site stated that the straw is only commercially compostable.^56^ To meet the standards, the certifying bodies prescribed a maximum nominal thickness for the straws of 246 μm (BPI) and 190 μm (TÜV). The measured thickness was greater than the specified limit for TÜV certification (Table 1). The polymer used in the NatureStar resin was undisclosed. No inorganic additive content was detected (Table 1). The IR spectrum for the Resin 1 straw suggested it was a PLA/PBAT blend, displaying peaks for both polymers (Figure S10).^58,59^ The IR spectrum of the Resin 1 straw included characteristic peaks for PLA at 1754 and 757 cm^–1^ and for PBAT at 1712, 1169, 1121, and 725 cm^–1^.

### Resin 2

According to marketing materials and the product Web site,^60^ the Resin 2 straw was made from SeaTech resin, a seaweed-derived biopolymer blend compounded with “shell powder” and “mineral color”. More descript details on the material were disclosed in published patents,^14,61^ wherein the formulated resin can be composed of at least κ-carrageenan, chitosan, glycerol, calcium carbonate, and water. The measured inorganic additive content was 30.8 ± 0.1 wt % (*n* = 3), and elemental analysis by XRF of the inorganic material detected K, Ca, Co, and Cr with relative abundances of 6.22% ± 1.12% (*n* = 3), 24.49% ± 5.98% (*n* = 3), 1.36% ± 0.51% (*n* = 3), and 1.07% ± 0.38% (*n* = 3), respectively. The IR spectrum of the inorganic material included peaks for CaCO_3_, as well as several others. Presumably, some of these additional IR peaks and the Co and Cr detected by XRF belong to the blue mineral-based colorant (possibly cobalt chromite blue). The potassium likely came from K_2_CO_3_, as it is mentioned as a possible component in one of the published patents, and no other elements were detected by XRF as possible anions.

### Biodegradation under Coastal Marine Conditions

Given that biodegradability is a marketing point for many, if not all, of the alternative drinking straws and the prevalence for drinking straws to leak into the marine environment, the marine biodegradation properties of the straws were evaluated. While short-term bottle incubations help establish whether microbes can degrade a polymer, open mesocosm systems provide more environmentally relevant evidence for microbial degradation of a polymer over other available carbon sources in coastal settings (e.g., natural organic matter). Thus, we incubated the straws for 30 weeks in a continuous flow seawater mesocosm to assess the mass loss rates of the straws under coastal seawater conditions. All the straws developed appreciable biofilms throughout the incubation (Figure 1A). By 30 weeks, the PHA straws lost 30–50% of their mass, the paper straw lost ∼70%, and the Resin 1 straw lost ∼10% (Figure 1B,C). The PP-based straws showed no mass loss (Figure 1C). Having established the potential for the biodegradability of several straws and that our mesocosm is without mechanical action and sunlight to cause fragmentation (Extended Materials and Methods in the Supporting Information), any mass loss was attributed to biodegradation.

**Figure 1:**
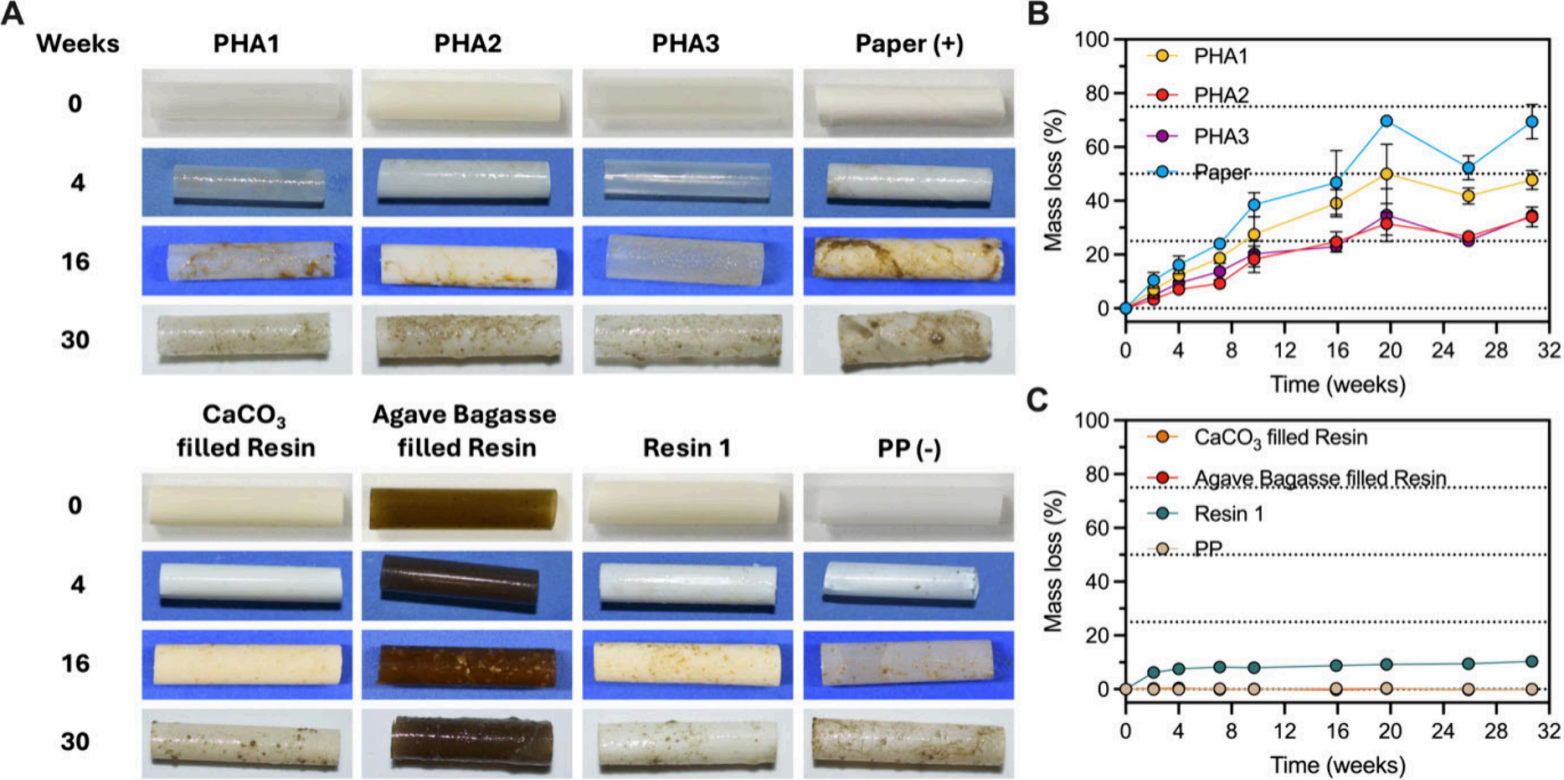
Representative images of each straw initially and after 4, 16, and 30 weeks in a flow-through seawater mesocosm (A). Relative mass loss of the PHA and paper straws (B) and the PP-based and Resin 1 straws (C). Data are presented as the mean ± standard deviation of three replicates unless otherwise noted (Table S4).

The Resin 2 straw broke apart in the mesocosm and developed a paste-like consistency, preventing an assessment of its mass loss. When incubated in the mesocosm, the straw broke apart and accumulated at the bottom of the tank within 8 weeks (Figure S11). Over the next 30 weeks, there was little visual change in the amount of the Resin 2 material that settled to the bottom (Figure S11). Presumably, the material that collected at the bottom of the tank and persisted was the inorganic material that made up the Resin 2 straw, as it retained its blue color.

As orthogonal measures of marine biodegradation to the mass loss measurements, the microbial communities associated with the straws were assayed for their oxygen respiration rates and characterized for their community composition. Respiration rates greater than a seawater-only baseline provide evidence that the associated microbes can biodegrade the straws. These short-term bottle incubations over 3 weeks showed that the PHA1–3, Resin 1, and paper straws stimulated microbial respiration to varying degrees above the baseline (Table S6), supporting marine biodegradability. The Resin 1 and paper straws had comparable respiration rates, about double those of the PHA1–3 straws. The PP-based straws (PP, CaCO_3_ filled Resin, and Agave Bagasse filled Resin straws) had respiration rates comparable to seawater, discounting their marine biodegradability. The Resin 2 straw was unable to be assayed due to complications with the poisoning agent (Section S2).

In contrast to the agreement between microbial respiration and mass loss for the PHA, paper, and PP straws, the Resin 1 straw displayed unique behavior (Figure 1C and Table S6). The Resin 1 straw had a relatively large amount of mass loss at the first time point, followed by a much slower mass loss rate for the remainder of the incubation (Figure 1C). This behavior is likely from the rapid leaching of a biodegradable additive,^14,15^ consistent with the large oxygen respiration rate for the straw measured in the short term respiration experiments.

Characterizing the microbial communities associated with the drinking straws can inform whether the microbes are using the material as a growth substrate or simply a surface for attachment. Beta diversity analysis by Bray–Curtis dissimilarity indicated distinct community compositions in response to the different materials (PERMANOVA, *p* < 0.01) (Figure 2), suggesting the degradability (Figure 1) and material properties of the straws selected for distinct microbial communities. The straws that degraded had unique microbial communities, differentiating by the type of material. In contrast, the three PP-based straws had similar community structures and were separate from those of the degradable straws and seawater. This result suggests that these straw materials mainly provided surfaces for the colonization of microorganisms rather than substrates for metabolism, an observation consistent with our previous findings.^19^ Microbial community composition analysis at the family level revealed detailed differences between the PHA, PP-based, Resin 1, paper straws, and seawater (Figure S12 and Section S3), paving the way for future investigations to delve into the details of specific microbial species and functions underlying plastic biodegradation in the ocean.

**Figure 2:**
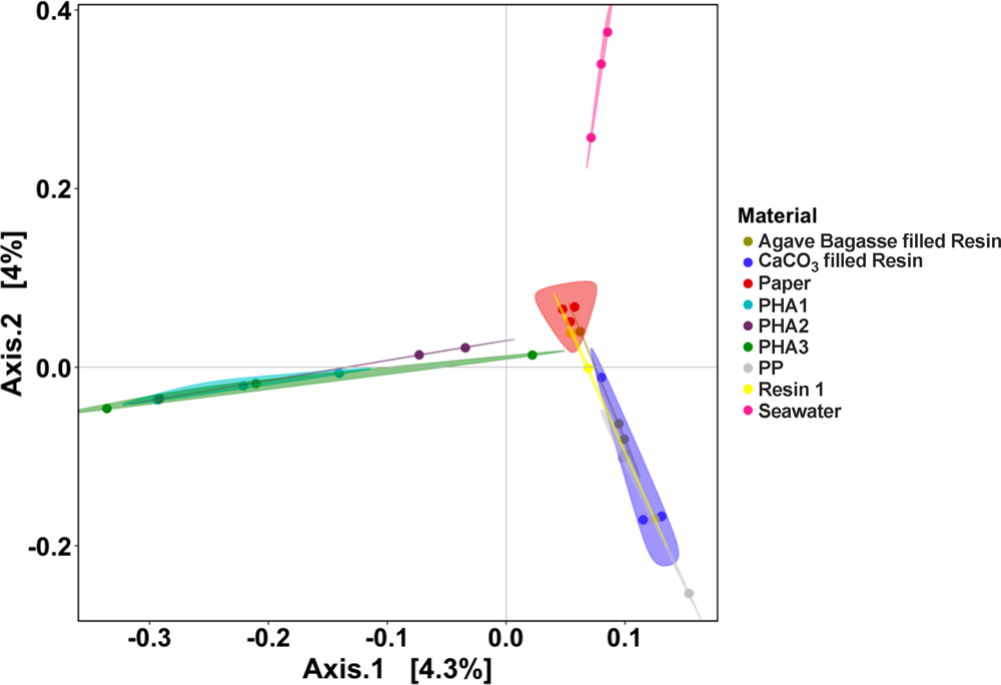
Beta diversity of microbial communities based on Bray–Curtis dissimilarity of 16S rRNA gene sequences visualized by principal coordinates analysis.

### Estimated Environmental Lifetimes under Coastal Marine Conditions

The mass loss data were fit to a phenomenological surface erosion model to derive the specific surface degradation rate (*k*_d_) of the straw materials. This material property quantifies the rate of surface erosion of a material in a given environment, accounting for the effects of material properties and environmental conditions. The values of *k*_d_ for the PHA straws were 50 ± 6 μm/year for PHA1, 54 ± 5 μm/year for PHA2, and 66 ± 8 μm/year for PHA3. Although minor, the differences in the values of *k*_d_ for the PHA straws suggested an effect of the polymer, which should be investigated further to understand the influence of PHA copolymer composition on degradation rates. The values of *k*_d_ for the paper and Resin 1 straws were 190 ± 18 and 7 ± 0.6 μm/year, respectively. The values of *k*_d_ for the PP-based straws were constrained to less than 0.5 μm/year.

With the values of thickness and *k*_d_, the projected environmental lifetimes of the straws were calculated. The paper straw had the shortest projected environmental lifetime of 11 ± 1 months. Values of projected environmental lifetime for the PHA straws were 15 ± 2 months for PHA1, 21 ± 2 months for PHA2, and 23 ± 3 months for PHA3. The Resin 1 straw had a projected lifetime of 197 ± 20 months. The projected environmental lifetimes for the PP-based straws were constrained to a minimum of 1800 months. No environmental lifetime was projected for the Resin 2 straw.

## Discussion

Drinking straws reflect the broader challenges to create products that (i) work as intended, (ii) meet consumer preferences, (iii) are inexpensive and profitable, and *now* (iv) satisfy circularity, sustainability, and degradability goals. Achieving these objectives for a drinking straw, an arguably simple product, is not so simple, as evidenced by the different straws investigated in this study. Each product satisfies these four criteria to varying degrees and via different approaches. For the first three criteria, each straw works as intended, meets consumer preferences, and is inexpensive to differing extents (Section S4).

To satisfy circularity, sustainability, and degradability goals, each straw approached them through different combinations of feedstock, plastic formulation, and EoL management. To semiquantitatively assess these approaches, an eco-audit framework was used that included sustainability metrics of renewability, material efficiency, embodied GHG emissions, embodied water usage, and EoL impacts. An eco-audit is often employed in material selection when precise data about a product or process is unknown (e.g., early on in engineering design or where specifics about the manufacturing of a product are limited) and comparison between choices is of interest.^16^ It is similar in nature to the life cycle assessment of technologies at early stage technology readiness levels.^62^ All but the Resin 2 straw was included in this analysis because of a lack of reliable data for this material. Nonetheless, a brief general discussion on the sustainability of seaweed-based materials is presented in Section S5. Additionally, the straws investigated in our previous study^14^ (CDA, PLA, PHA4, and PHA5; see Section S1) were included to reflect the broader design space of drinking straws. Primary research and experimentally determined biodegradation rates for these four other straws are included in Section S1 and Table S2.

### Renewability

To fit into a circular economy framework, a renewable feedstock is one in which the carbon has been recirculated, including carbon obtained from biomass, industrial byproducts, waste CO_2_ or CH_4_, or recycled plastics.^63^ Most straws were made from 100% renewable materials (Figure 3A). Only those with fossil-based polymers (i.e., PP and Resin 1) were partially or entirely made from nonrenewable materials.

**Figure 3:**
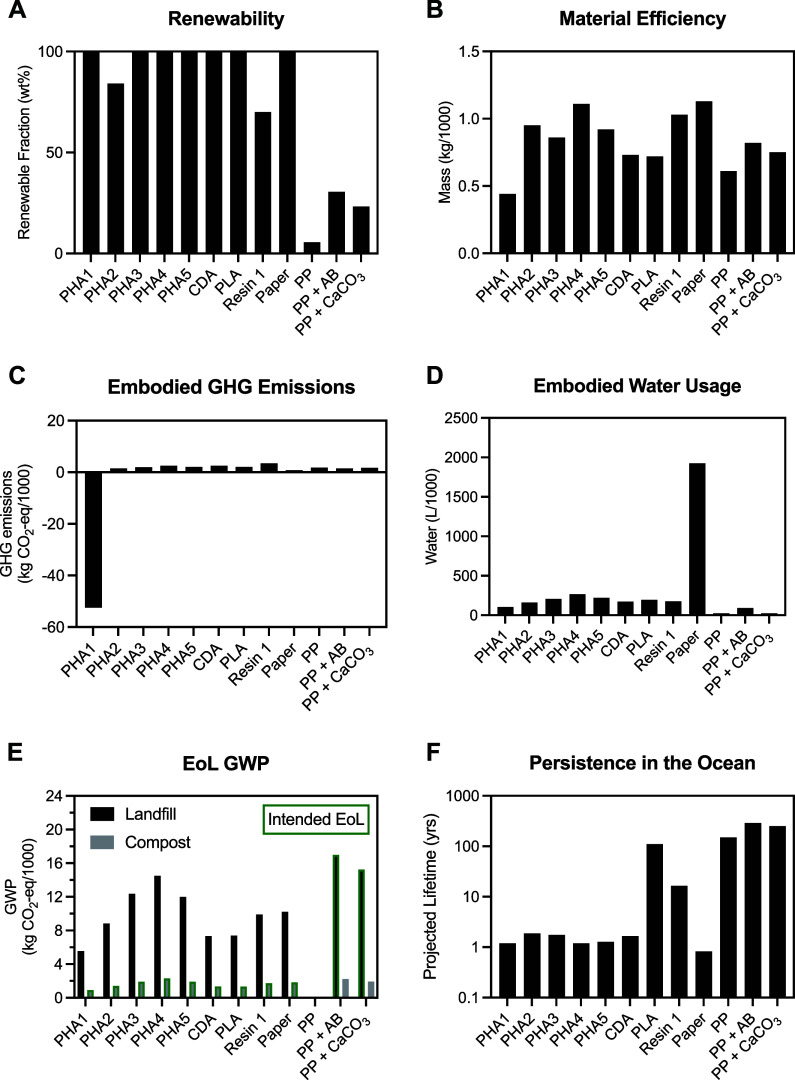
Comparison of sustainability metrics: renewability (A), material efficiency (B), embodied GHG emissions (C), embodied water usage (D), EoL GWP (E), and persistence in the ocean (F). The Agave Bagasse filled Resin and the CaCO_3_ filled Resin are abbreviated as PP + AB and PP + CaCO_3_, respectively. Note that the y-axis of (F) is on a log-scale.

The investigated PP-based straws appear to use comparable amounts of polymer to that of a pure PP straw, negating the otherwise would be benefit of the renewably sourced filler. PP is primarily produced using nonrenewable petroleum sources, and ∼5.5% is recycled.^16^ Biogenic fillers (i.e., oyster-derived CaCO_3_ and agave-derived bagasse) can, in principle, reduce the total amount of polymer by replacing it with a renewable material. However, the CaCO_3_ filled Resin was ∼18 wt % CaCO_3_, meaning that ∼612 mg of the straw was PP, effectively the same amount of material as the pure PP straw (∼615 mg).

PHAs are produced by fermentation, using various carbon sources as feedstocks.^64^ The PHA used in the PHA1 straw uses CH_4_ diverted from anthropogenic sources (e.g., wastewater, cattle farming, landfills, and coal mining) as the carbon source, yielding a carbon offsetting material. While the C in PHA1 was determined to be from a fossil-derived source, the recirculation of CH_4_ by a carbon capture method into P3HB polymer makes the material renewable.^63^ In contrast, the PHA2–5 straws use agricultural products, such as vegetable oils, to produce their PHA materials. PHA2 was not 100% renewable because of its fraction of CaCO_3_ of unknown origin (assumed nonbiogenic).

Similar to PHAs, PLA is synthesized using lactic acid produced by the fermentation of agricultural products.^65^ Therefore, PHAs and PLA made using these feedstocks can be certified as 100% biobased. The Resin 1 straw was determined to be a blend of PLA and PBAT, and so was partially biobased, due to its PLA content. PBAT is predominantly fossil-based, but efforts are being made to circularize its feedstocks to include those from recycled materials and biobased sources.

Historically, CDA has been a mixed feedstock wherein the cellulose was bioderived, and the carbon within the acetyl modification was derived from coal. Recognizing the nonrenewability of coal as a carbon source, more sustainable grades of CDA are being produced by deriving the acetyl C from molecularly recycled mixed waste.^66^

### Material Efficiency

Material efficiency quantifies the amount of material used to make a functional product.^67^ Optimizing the material efficiency of a plastic product has the added benefit of reducing many other environmental impacts (e.g., GHG emissions and freshwater usage) because these impacts are largely proportional to the amount of material used.^68–70^ Material efficiency also affects environmental impacts related to shipping because fuel efficiency decreases with increasing cargo weight by a nontrivial amount.^67,71^ Because all the straws have the same function, their mass is a measure of their material efficiency. The PHA1 straw had the greatest material efficiency, followed by the PP straw (Figure 3B). The PHA1 straw used ∼33% less material than the PP straw. The other PHA and PP-based straws were less materially efficient, using more material to produce a functional product. The paper straw was one of the least efficient, requiring ∼60% more material than the PP straw.

### Embodied GHG Emissions

A first estimate for the GHG emissions of a product are the emissions associated with the production of its materials. Because the specific embodied GHG emissions of plastics are within an order of magnitude of each other, as were the masses of each straw, differences in GHG emissions between products were expected to be marginal.^30^ Every straw, except for PHA1, was carbon-positive, ranging from 0.80 kg CO_2_-eq/1000 straws for the paper to 3.45 kg CO_2_-eq/1000 straws for the Resin 1, PLA/PBAT-based straw (Figure 3C).

PHA1 was the only straw with net-negative embodied GHG emissions (Figure 3C), due to diverted CH_4_ emissions as its feedstock. Scaling this approach could impact the global C cycle and climate change. For instance, if all the C used in plastic packaging globally (∼142 Mt/yr^72^) were P3HB (∼56 wt % C) and came from anthropogenic CH_4_, then ∼106 Mt CH_4_ (∼3.0 Gt CO_2_-eq) would be needed. Adopting this strategy would offset ∼30% of the ∼9.7 Gt CO_2_-eq of annual global anthropogenic CH_4_ emissions,^73^ thereby highlighting the promise of scaling this design strategy.

Relative to selecting materials with more renewable feedstocks, the effectiveness of biogenic fillers appears negligible. The theoretical maximum offset in GHG emissions for oyster-derived CaCO_3_ is ∼0.44 kg CO_2_-eq/kg CaCO_3_. Based on its formulation, the theoretical maximum reduction in embodied GHG emissions compared to the PP straw is only 4% for the CaCO_3_ filled Resin straw. The value of the theoretical specific embodied GHG emissions for agave-derived bagasse is greater than oyster-derived CaCO_3_, offsetting ∼1.51 kg CO_2_-eq/kg agave-derived bagasse. In practice, these values are likely less because of GHG emissions associated with processing the materials. While the amount of agave-derived bagasse used in the Agave Bagasse filled Resin straw was not disclosed, biocomposites of agave-derived bagasse and PP typically include 20–30 wt % bagasse.^22–24^ Within this range, the estimated reduction in embodied GHG emissions for the Agave Bagasse filled Resin straw compared to the PP straw was 8 to 28%.

### Embodied Water Usage

An impact of growing concern is a material’s embodied water usage because of the scarcity and diminishing quality of freshwater resources globally.^74,75^ Generally, plastics derived from agricultural products have greater embodied water usage than petroleum-derived plastics, due to the water used in agriculture and downstream processing. However, these impacts can be an order of magnitude less than those resulting from paper production. Paper and pulp mills pollute freshwater ecosystems and diminish their value, as well.^76^ Reported values for the embodied water usage of PP, PHA, and paper are 32, 240, and 1700 L/kg, respectively.^16^ Thus, while the paper straw can have some of the lowest embodied GHG emissions (∼2–3 times less than the others, excluding PHA1), it can have substantial embodied water penalties (∼10–80 times more than the others) (Figure 3D). Water is a valuable natural resource; thus, reducing its usage as much as possible will benefit society.^77,78^

### EoL (Mis)­management and GWP

#### Managed EoL

The strategies advertised and promoted for the managed EoL of the straws varied with the material. The PP-based straws were intended to follow a linear EoL path with disposal in landfills. The PHA-based, paper, and Resin 2 straws were intended to follow a more circular EoL path with disposal in industrial or home composting systems. The Resin 1 straw was intended to be industrially composted.

Today, most managed plastic waste is landfilled,^79^ some is incinerated, and even less is recycled and composted.^80^ While PP is recyclable, PP straws are often not accepted by municipal recycling facilities because of their size and shape, and contamination from food. In the U.S., ∼ 63% of municipal solid waste is landfilled.^81^ In many instances, it is the only waste management option available.

The filled PP straws were claimed to biodegrade in landfills. The CaCO_3_ filled Resin straw was advertised for disposal in landfills and to be formulated with an enzymatic package to support its biodegradation in that environment.^50^ These additives are designed to stimulate enzymatic degradation of the polymer by fouling microbes under anaerobic conditions, though their effectiveness has been dubious.^82,83^ Collectively, the respiration, mass loss, and microbial community composition analyses suggest these additives were not stimulating the biodegradation of the PP matrix in aerobic marine conditions. The Agave Bagasse filled Resin straw was advertised for “avoiding landfills” but did not mention where it was intended to go. Being PP, this straw likely could only be properly disposed of by landfilling or incineration. There is little evidence to support the possibility of either straw’s biodegradation in landfills (i.e., no third-party certificates for the products). However, taken at face value, assuming the claims hold that the filled PP straws can biodegrade in terrestrial and landfill environments, their disposal in landfills would result in biogenic CH_4_ emissions (Figure 3E). Landfills contribute ∼10% of global anthropogenic CH_4_ emissions, and their leachates can diminish fresh and groundwater resources.^84^ Because of biogenic CH_4_ emissions, promoting landfilling as the managed EoL strategy can have significant associated GHG emissions for all but the nonbiodegradable straws (Figure 3E).

Composting can be an alternative EoL strategy. Unlike landfilling, composting is an aerobic microbial degradation process that converts organic matter to biomass and CO_2_, not CH_4_. As a result, the GWP can be an order of magnitude less for compost than landfill (Figure 3E). Composting can be operated on the industrial scale using elevated temperatures or at the individual scale using ambient conditions (i.e., home composting). Some materials are only certified industrially compostable (e.g., PLA), while others are certified home-compostable (e.g., PBAT, PHA, and CDA). For food-soiled, single-use items, composting affords a strategy well-equipped to handle such waste alongside food scraps.

Nonetheless, composting has its challenges. Presently, composting is rarely practiced and lacks available infrastructure at the municipal level. In the U.S., it has been estimated that only ∼4% of food waste is composted.^85^ Thus, while products are meant for and designed to be composted, they may rarely follow that intended path and instead enter landfills or incinerators. Moreover, composting is not without its environmental impacts, as well. Improperly aerated compost can result in CH_4_ emissions in the same way as landfills. For composting to be broadly adopted, it will require greater investment by consumers and communities in composting capabilities for biodegradable plastics.

#### Mismanaged EoL

Drinking straws can be mismanaged at their EoL by being littered or disposed of in the wrong waste stream (e.g., landfilled instead of composted). In the event of littering, several of the straws will persist in the coastal ocean despite claims of their degradability in other environments (e.g., compost or soil). The PP-based and Resin 1 straws were estimated to persist for decades or much longer if leaked (Figure 3F). Notably, the Resin 1 straw exhibited ∼10% mass loss after 30 weeks of incubation (Figure 1). However, the microbial community structure was similar to that of the PP-based straws (Figure 2), which is unexpected if the community was adapting to metabolize the polymer blend. Considering this finding and those of others investigating PLA/PBAT blends,^86^ further analyses of the Resin 1 material are necessary to confirm whether the shallow mass loss rate after the first time point was due to a residual additive slowly leaching, the slow metabolism of the polymer by fouling microbes, or other processes. Conversely, because the PHA- and CDA-based straws were shown to biodegrade in the coastal ocean, they were estimated to last 1–2 years, slightly longer than paper. For the straws that were not persistent, their differences in estimated environmental lifetimes reflected trade-offs in material and geometric properties.

Disposal of drinking straws in landfills intended for compost results in additional and more potent GHG emissions that otherwise would not have been produced. This outcome is because anaerobic landfill conditions produce CH_4_ during the biodegradation of the material that escapes collection. The added GWP from improper disposal of compostable materials in landfills was estimated to range from 4.64 to 13.44 kg CO_2_-eq/1000 straws (Figure 3E), resulting in approximately six times more GWP per straw. The conventional PP straw, in contrast, has zero GWP in compost or landfills because of its presumed recalcitrance in these environments.

### Social Costs of GHG Emissions and Plastic Pollution

Social costs were used to quantify, contextualize, and compare the environmental impacts of marine plastic pollution and GHG emissions. Social costs reflect the external costs to society associated with a good, service, or outcome. The exchange constant (the conversion factor of environmental impact to monetary value) for the social cost of marine plastic pollution was proposed to range between 4.58 to 45.8 US$/year kg plastic.^87^ Using the lower value for a conservative estimate, switching from persistent to nonpersistent plastics for drinking straws can equate to savings for society of ∼3 US$/1000 straws (Table S7). Scaled to the estimated number of straws that leak into the environment in the U.S. annually, the switch represents a potential savings of ∼150 million US$/year.

Social costs were also quantified for GHG emissions associated with producing straws and improper disposal of compostable straws in landfills. The exchange constants for social costs of CO_2_ and CH_4_ have been estimated to be 0.22 US$/kg CO_2_ and 3.40 US$/kg CH_4_, respectively.^88,89^ The social costs for the embodied GHG emissions of the straws (excluding PHA1) ranged from 0.18 to 0.92 US$/1000 straws and scaled to the annual consumption rate of drinking straws in the U.S. could cost 9–46 million US$/year. PHA1 was unique because it had negative embodied GHG emissions at the beginning of life from using diverted anthropogenic CH_4_ as a feedstock. As a result, it was estimated to equate to savings of 11.55 US$/1000 straws or ∼578 million US$/year. The social costs from the added GHG emissions due to landfilling a compostable drinking straw ranged from ∼0.21 to 0.61 US$/1000 straws (Table S7) and scaled to the estimated annual U.S. drinking straw consumption rate, representing a potential burden of 11–31 million US$/year.

From this analysis, three key points emerged. First, conventional PP straws do not contribute to societal costs in terms of GHG emissions at EoL regardless of whether they accumulate in compost or landfill because they are nonbiodegradable; however, they contribute sizably to societal costs when littered in the marine environment for that same reason. Second, for PHA1, the societal costs at EoL, regardless of the scenarios investigated, could be entirely offset by the savings to society by using diverted CH_4_ emissions as a feedstock to produce the polymer. Lastly, the added societal costs from the improper disposal of compostable and marine-biodegradable straws (i.e., paper, PHA, and CDA) in landfills (i.e., the difference between the landfill EoL GWP and compost EoL GWP) were estimated to be 18–36 times greater than the social costs from their improper disposal as marine litter (Table S7). While this analysis only discusses the social costs of two environmental impacts (plastic pollution and GHG emissions), of which there are several others (e.g., resource utilization and eutrophication), it was instructive for contextualizing the relative burden of the different drinking straws at EoL.

### Limitations

The outcomes of the eco-audit are not without limitations and caveats. It should be reiterated that the eco-audit is largely based on average literature values, which are useful for semiquantitative comparison. The GWPs were estimated using a theoretical equation and actual values will vary with time and local conditions. For example, reports on the anaerobic biodegradation of PLA and PBAT in landfills have been variable,^90–93^ and thus the values presented for them should be interpreted as an upper theoretical value. Likewise, the actual environmental lifetimes for the straws will vary with environmental conditions (e.g., temperature^86^ and location^94^). Moreover, for those materials that do not biodegrade in the ocean,^14,15^ but nonetheless experience hydrolysis (e.g., PLA^95^), the projections of their environmental lifetimes are less confident. PLA can be expected to persist for decades in the ocean based on estimated hydrolysis rates;^95^ which is still much longer than those of more readily biodegradable materials. Multiyear studies of PLA products in marine settings are necessary to constrain this potential uncertainty.^96^ Additionally, ecotoxicity was not directly included in the eco-audit, which may reveal differences between the polymers, particularly over their lifecycle.^97–100^ Building on this point, plastics are composed of polymers and additives.^101^ The impact of small molecule organic additives was not considered in the eco-audit and may sway the outcomes.^101,102^ The development of new eco-audit frameworks for material selection will be necessary to address the trade-offs between function and ecotoxicity.

### Implications

From this case study of drinking straws, four valuable lessons were learned. First, the combination of approaches taken by the PHA1 straw resulted in a product that qualitatively and quantitatively could be the least impactful. It fits within a circular materials framework, had the greatest material efficiency, was nonpersistent in the coastal ocean, and was carbon–offsetting by utilizing diverted anthropogenic CH_4_ emissions as a feedstock. These design decisions are a promising set that could be adopted for other materials and products. Second, inorganic fillers can offset GHG at EoL, and biogenic wastes can be repurposed as fillers into new products, sequestering carbon as well. However, merely adding fillers to the status quo polymer (e.g., PP) will likely not produce significant benefits for the environment compared to using an alternative polymer. Third, many of the claims made by brands about circularity had limited supporting data, which likely will lead to the mismanagement of their products and unreliable outcomes. Thus, additional or a set of required certifications are needed to give credibility to life cycle claims and the impacts that can result from different life cycle scenarios. In this way, much like a nutrition facts label, consumers could evaluate the sustainability and environmental impacts of products. Lastly, improper disposal of compostable products in landfills can have sizable environmental impacts, thereby warranting greater investment in infrastructure to manage compostable plastics and associated food waste properly. Otherwise, many of the advantages afforded by these materials will be nullified. Moving forward, from our analysis, consumers, brands, and policymakers should support products that divert anthropogenic GHGs for use as renewable feedstocks, promote the design of products for material efficiency and minimal persistence, and bolster proper disposal of compostable products by enhancing consumer education and supporting investment in industrial and home composting facilities.

## Supplementary Material



## Notes

Extended materials and methods, details of previously investigated drinking straws (Section S1), interference by the poisoning agent (Section S2), microbial community composition analysis at the family level (Section S3), discussion of nonsustainability-related design considerations for drinking straws (Section S4), discussion of the sustainability of seaweed-based materials (Section S5), plots of relative mass loss data for each straw with curve fit (Figure S1), random samplings of straw thickness, *k*_d_, and projected environmental lifetimes (Figures S2–S6), IR spectra of select drinking straws (Figures S7–S10), photographs of Resin 2 in the mesocosm (Figure S11), microbial community compositions at the family level (Figure S12), list of drinking straws on the market (Table S1), properties of previously investigated drinking straws (Table S2), mass loss data (Table S3), relative mass loss data (Table S4), global warming and resource utilization properties (Table S5), short-term microbial respiration rates (Table S6), calculated social costs (Table S7), and additional references (PDF)
