# Supplementary material for: Strategies for Designing Circular, Sustainable, and Nonpersistent Consumer Plastic Products: A Case Study of Drinking Straws
Source: Environ Sci Technol. 2025 Aug 22;59(34):18177–89. doi: 10.1021/acs.est.5c05448 (PMC12409878; doi:10.1021/acs.est.5c05448)
Supplement: Supplementary file 1 [file es5c05448_si_001.pdf]

Supporting Information

Strategies for designing circular, sustainable, and non-persistent consumer plastic products: a case study of drinking straws

Bryan D. James[1,2,*], Yanchen Sun[1], Kali Pate[1], Brenden Irving[1], Collin P. Ward[1,*]

[1]Department of Marine Chemistry and Geochemistry, Woods Hole Oceanographic Institution, Woods Hole, MA, USA 02543

[2]Department of Chemical Engineering, Northeastern University, Boston MA, USA 02115

*Correspondence: b.james@northeastern.edu; cward@whoi.edu

Number of Pages: 32
Number of Figures: 12
Number of Tables: 7

Extended Materials and Methods

Material characterization

The straws were characterized by several different approaches to fill in the gaps in the information provided by their manufacturers. The density of each straw was determined from measurements of their geometry and mass. Pieces of each straw were cut to have simple geometries (i.e., rectangles). The geometric dimensions (length, width, and thickness) of each piece were measured with digital calipers (Mitutoyo CD-6" ASX; uncertainty of 0.02 mm, resolution of 0.01 mm) and used to calculate the volume of the piece. The mass of each piece was measured with an analytical balance (Mettler Toledo AG245; readability of 0.1 mg; repeatability of 0.1 mg). The density of each piece was calculated as the ratio of the measured mass and volume. The inorganic additive content of each drinking straw was measured by loss-on-ignition.[1,2] Briefly, ~500 mg of each drinking straw was placed in a pre-massed, pre-combusted ceramic crucible and combusted in a muffle furnace at 450 ºC for 8 h. The remaining ash was then re-massed. The ash of the PHA2 and Resin 2 straws were screened for their elemental composition using an Oxford Instruments X-MET 7500 hand-held X-ray fluorescence (XRF) device.[2] The straws for which their resin was undisclosed (the $CaCO_3$ filled Resin, Agave Bagasse filled Resin, and Resin 1 straws) were evaluated by attenuated total reflectance-Fourier transform infrared spectroscopy (ATR-FTIR). IR spectra were collected using an Agilent Cary 630 FTIR with a diamond crystal ATR module, averaging 32 scans with 2 cm^{-1} resolution. When necessary, spectra were processed in Open Specy,[3] applying a linear baseline and first-order smoothing, and polymer identity was assigned based on comparison to the Open Specy database. To confirm the carbon source of the PHA1 straw, measurement of its stable and radiocarbon isotopic composition were made by the National Ocean Science Accelerator Mass Spectrometry (NOSAMS) facility using elemental analysis.[4,5]

Oxygen respiration

Microbial respiration of all straws was measured via dissolved oxygen (O_2) consumption in short-term, closed-system incubations.[6,7] Pieces of each straw ~13 mm in length were placed in 12 mL exetainer vials and filled to no headspace with unfiltered seawater pumped to the Environmental Systems Laboratory (ESL) from Martha's Vineyard Sound, MA, USA (41° 31' 52.0" N, 70° 38' 36.6" W). Seawater-only controls were prepared, as well. The vials were incubated in the dark at room temperature for three weeks. Samples for four time points were prepared in triplicate for each straw, including an initial time zero set of samples. At specified time points (T0, T1, T2, and T3), the vials in triplicate for each straw were poisoned with 50 µL of saturated $HgCl_2$ to arrest microbial activity and stored in the dark at room temperature until analysis. All vials were analyzed for their O_2 concentration using a Bay Instruments membrane inlet mass spectrometer at the end of the three weeks.[7] The O_2 concentration was used to calculate the seawater-only corrected respiration rates by linear regression. For the Paper and Resin 1 straws, only the T0 and T1 were used for the regression analysis because the vials went anoxic by T1. For the PHA1-3 straws, only T0, T1, and T2 were used for the regression analysis because the vials went anoxic at T3. All time points were included for the PP-based straw.

Continuous flow natural seawater mesocosm

Continuous flow natural seawater mesocosms were used in the ESL. The mesocosms included a 380 L aquaria tank supplied with a continuous flow of seawater by a head tank. Martha's Vineyard Sound seawater was pumped to the ESL, tempered to 20 ºC, collected in a head tank, and flowed to the mesocosm tank with an average flow rate of 190 L/hr, yielding a residence time of ~120 minutes. Details of the seawater pumping, filtering, and temperature tempering system have been described previously.[5]

Mass loss measurements

Samples were ~25.4 mm segments cut from each straw. This length was chosen because of size constraints to fit samples into the mesocosm tank, and it achieved a starting mass of at least ~50-100 mg for each straw material. This initial mass ensured that the accuracy for mass measurements by our analytical balance (Mettler Toledo AG245; readability of 0.1 mg; repeatability of 0.1 mg) would be ≤0.5% for the duration of the time series. The same analytical balance was used for the entirety of the time series. All samples were massed before placement in the mesocosm tank.

Straw samples were suspended ~10 cm from the bottom of the mesocosm tank on stainless steel wire holders, each holding four samples. Samples were suspended rather than clamped because, in trial experiments, clamping led to preferential cracking of some straws at points of deformation. Samples were randomly positioned on the sample holders, and time points were randomly arranged in the tank along its long axis to mitigate any potential bias of sample positioning in the tank.

Samples were collected as sets at prescribed time points by removing a random sample holder from the tank and cutting the sample holder to liberate a sample. Each sample was photographed and placed into pre-weighed 2 mL PP microfuge tubes filled with MilliQ water and incubated for ~30 minutes. After the incubation, samples, and tubes were lightly rinsed with copious amounts of MilliQ water to remove detritus. Then, samples in their respective tubes were placed open to dry at 60 ºC for 48 hrs in a Fisher Scientific IsoTemp 637G oven. Samples in their tubes were then removed from the oven, closed, allowed to return to room temperature, and massed.

Each straw at each time point was evaluated for mass loss in triplicate (**Table S3**). Mass loss was calculated as the relative mass loss (%) being the difference between the initial mass of the sample (m_0) and the mass of the sample (m_t) at the time point (t) normalized to the initial mass of the sample (**Equation S1**).

$$Mass\ loss\ (\%) = \frac{m_0 - m_t}{m_0}(100\%) \tag{S1}$$

Surface erosion model

The relative mass loss data was analyzed using a phenomenological surface erosion model[8,9] (**Equation S2**) in which $\frac{\partial m}{\partial t}$ is the change in mass with time, m is the instantaneous mass, k_d is the specific surface degradation rate, A_s is the surface area, and V is the volume.

$$\frac{\partial m}{\partial t} = -mk_d\frac{A_s}{V} \tag{S2}$$

Equation S2 was solved for a hollow cylinder of initial length l_0 and initial thickness h_0 and shifted and scaled using a constant, β, to account for mass loss due to leachable components (e.g., plasticizer) or other initial jumps in mass loss between the initial mass and the first time point to yield **Equation S3**. Accordingly, the trivial initial data point of zero mass loss at time zero was not included in any model fitting.

$$\frac{m(t)}{m_0} = 100\%\left[\left(1 - \frac{(l_0 - 2k_d t)(h_0 - 2k_d t)}{l_0 h_0}\right)(1-\beta) + \beta\right] \tag{S3}$$

l_0 was assumed to be 25.4 mm for each sample, which is valid because **Equation S3** is largely insensitive to changes in l_0 when l_0>>h_0 as is the case for the drinking straw samples.

Regression and bootstrapping

The relative mass loss data (**Table S4**) was fit to **Equation S3**, using nonlinear least-squares regression in GraphPad Prism 10.1.0 (264) to evaluate the model's goodness of fit for each straw (**Figure S1**). All fits had R^2 >0.75, the residuals satisfied normality assumptions as determined by the D'Agostino-Pearson omnibus normality test for all but PHA1 and PHA3, and the residuals were homoscedastic for all but Resin 1. k_d and its uncertainty was computed by a combination of bootstrapping and nonlinear least-squares regression in MATLAB R2020b by randomly sampling a value for the straw thickness (h_0) from a normal distribution defined by the mean and standard deviation of the measured thickness (**Table 1**) and fitting the relative mass loss data (**Table S4**) using the sampled value for thickness. The random sampling and fitting process was repeated 5000 times, during which, for each fit, a value of k_d was sampled from a normal distribution defined by the respective fit k_d and its standard error. Thus, 5000 values of k_d were generated based on the uncertainty of the thickness measurement and the relative mass loss data. Similarly, 100,000 values of projected environmental lifetime (t_L) were generated for each straw according to **Equation S4**. This process was carried out by random sampling 100,000 values of k_d and h_0 from a normal distribution defined by their mean and standard deviation (**Figures S2-S6**). Increasing the sampling number by ten times did not change the mean and variation of sampled h_0, k_d, and t_L.

$$t_L = \frac{h_0}{2k_d} \tag{S4}$$

The relative mass loss data for the $CaCO_3$ filled Resin, Agave Bagasse filled Resin, and PP straws were not fit because any mass loss was within the uncertainty of the mass loss measurements. Instead, because no degradation was detected for these straws under the experimental conditions, the values of k_d were constrained to a maximum of 0.5 μm/year, representing the lowest measure of mass loss that is quantifiable for the sample dimensions used in these experiments and consistent with a previously reported value for PP.[10]

16S rRNA gene amplification, sequencing, and bioinformatics analyses

Straw and seawater samples were collected at week 17 from the continuous flow natural seawater mesocosm to capture microbial community composition. These samples were subjected to DNA extraction using a Qiagen DNeasy PowerBiofilm kit according to the manufacturer's protocol. Amplicon libraries for variable region V4 were prepared following previously established procedures.[11] The pooled amplicon libraries were sequenced using a 2×250 bp Illumina MiSeq platform at the Institute for Genome Sciences at the University of Maryland. Raw amplicon reads were processed in R (4.0.2) using the DADA2 (1.16) pipeline[12] for quality control, merging sequences, and assigning amplicon sequence variants (ASVs). Taxonomy was assigned to the family level using the naïve Bayesian classifier method against the SILVA v138.1 database.[13,14] Statistical analyses and plotting were performed using R. Beta-diversity was calculated using Bray–Curtis dissimilarity and visualized using the principal coordinate analysis (PCoA) plot in R with packages ggplot2[15] and phyloseq.[16] Statistical differences in microbial communities among different materials and seawater were determined using permutational multivariate analysis of variance (PERMANOVA) in R with package vegan.[17] The 16S rRNA gene amplicon sequencing data generated in this study were deposited in the European Nucleotide Archive under project PRJNA1162125.

EoL GWP Calculations

Agave Bagasse. Because the Buswell equation depends on the carbon, hydrogen, and oxygen content of the organic compound, the emissions for agave bagasse were calculated based upon its component contributions assumed only to come from its cellulose, hemicellulose, and lignin content, being 17.4, 59.6, and 13.6 wt% (kg component/kg agave bagasse), respectively.[18] Cellulose was approximated as $C_6H_{10}O_5$, yielding 3 mols CO_2 and 3 mols CH_4. Hemicellulose was

approximated as $C_5H_8O_4$, yielding 2.5 mol CO_2 and 2.5 mol CH_4. Lignin was approximated as $C_9H_{10}O_4$, yielding 4.25 mol CO_2 and 4.75 mol CH_4. The carbon content of cellulose, hemicellulose, and lignin are approximately 44, 40, and 65 wt% (kg C/kg component), respectively.[19] Thus, in landfill conditions, 1 kg of agave bagasse results in the generation of 0.44 kg CO_2 and 0.05 kg CH_4 from its cellulose content, 0.44 kg CO_2 and 0.16 kg CH_4 from its hemicellulose content, and 0.15 kg CO_2 and 0.06 kg CH_4 from its lignin content, yielding a 100-year GWP of 8.59 kg CO_2-eq/kg agave bagasse. The 100-year GWP in compost was calculated to be 1.48 kg CO_2-eq/kg agave bagasse.

PP. The value of χ_C was calculated according to the mer unit of PP (C_3H_6). While PP is conventionally considered recalcitrant to biodegradation, the manufacturers of the filled PP-based straws claimed biodegradation of the items. Thus, for those materials in landfill conditions, 1 kg of PP generates 0.79 kg CO_2 and 0.86 kg CH_4, yielding a 100-year GWP of 24.87 kg CO_2-eq/kg PP. The 100-year GWP in compost was calculated to be 3.14 kg CO_2-eq/kg PP.

P3HB. The value of χ_C was calculated according to the mer unit of P3HB ($C_4H_6O_2$). Thus, in landfill conditions, 1 kg of P3HB generates 0.90 kg CO_2 and 0.42 kg CH_4, yielding a 100-year GWP of 12.66 kg CO_2-eq/kg P3HB. In compost, the 100-GWP was calculated to be 2.05 kg CO_2-eq/kg P3HB.

PHBH. The value of χ_C was calculated according to the mer unit of PHBH ($C_4H_6O_2$)($C_6H_{10}O_2$). Thus, in landfill conditions, 1 kg of PHBH generates 0.88 kg CO_2 and 0.48 kg CH_4, yielding a 100-year GWP of 14.32 kg CO_2-eq/kg PHBH. The 100-year GWP in compost was calculated to be 2.20 kg CO_2-eq/kg PHBH.

PLA. The value of χ_C was calculated according to the mer unit of PLA ($C_3H_4O_2$). Thus, in landfill conditions, 1 kg of PLA generates 0.92 kg CO_2 and 0.33 kg CH_4, yielding a 100-year GWP of 10.25 kg CO_2-eq/kg PLA. The 100-year GWP in compost was calculated to be 1.83 kg CO_2-eq/kg PLA.

CDA. The value of χ_C was calculated according to the mer unit of CDA ($C_{20}H_{26}O_{14}$). Thus, in landfill conditions, 1 kg of CDA generates 0.94 kg CO_2 and 0.32 kg CH_4, yielding a 100-year GWP of 10.04 kg CO_2-eq/kg CDA. The 100-year GWP in compost was calculated to be 1.83 kg CO_2-eq/kg CDA.

PBAT. The value of χ_C was calculated according to the mer unit of PBAT ($(C_8H_6O_4)_m(C_6H_{10}O_4)_n(C_4H_{10}O_2)_{(m+n)}$). Thus, in landfill conditions, 1 kg of PBAT generates 0.90 kg CO_2 and 0.39 kg CH_4, yielding a 100-year GWP of 11.77 kg CO_2-eq/kg PBAT. The 100-year GWP in compost was calculated to be 1.97 kg CO_2-eq/kg PBAT.

Paper. Paper was assumed to be entirely cellulose, which was approximated as $C_6H_{10}O_5$. Thus, in landfill conditions, 1 kg of paper generates 0.81 kg CO_2 and 0.29 kg CH_4, yielding a 100-year GWP of 9.02 kg CO_2-eq/kg paper. In compost, the 100-year GWP was calculated to be 1.61 kg CO_2-eq/kg paper.

Section S1. Review of marketing, regulatory, and third-party certification documents and databases for previously investigated straws[20]

According to the packaging of the PHA4 and PHA5 straws, they were made from a PHA material (Trade name: Nodax) produced by Danimer Scientific. The PHA4 straw met several third-party biodegradation standards for compostability (Tüv Austria Home S1328; BPI Certified).[21,22] To meet the standards, the certifying bodies prescribed a maximum nominal thickness for the straw of 336 µm (Tüv) and 178 µm (BPI). The PHA5 straw was not third-party certified for compostability and instead advertised that the material was certified as ASTM D5988, ASTM D5271, ASTM D6691, ASTM D6400, ASTM D6866, EN 29408, and EN 13432:2002 compliant.

CDA-based straws are increasingly sold by U.S. retailers. One of the prominent converters is Stone Straw, which sells CDA straws under the brand name "Back to Earth".[23] The CDA straw met several third-party biodegradation standards for compostability (Tüv Austria Home S2714; BPI Certified).[21,22] To meet the standards, the certifying bodies prescribed a maximum nominal thickness for the straw of 185 µm (Tüv) and 200 µm (BPI).

Polylactide polymers have been U.S. Food and Drug Administration (FDA) approved for food contact since 2002.[24] At the time of approval, the notification to Cargill Dow (now Nature Works) raised the issue of littering and the distinction between degradation in municipal composting and the environment. Degradation in littered environments (e.g., roadsides) was not addressed in the filing with the FDA, unlike municipal composting, which was claimed.[25] The Plasticless PLA straw investigated by James et al.[20] did not advertise any compostability certifications and only stated a breakdown time of 6-12 months without specifying the environmental conditions for it. In contrast, other PLA drinking straw brands (e.g., EcoProducts) meet several third-party biodegradation standards for compostability (Tüv Austria Industrial S0685; BPI Certified) with the certifying bodies prescribing a maximum nominal thickness for the straws of 336 µm (Tüv) and 212 µm (BPI).[21,22]

Section S2. Interference by poisoning agent (mercuric chloride) during O_2 respiration measurements of Resin 2

Upon addition of mercuric chloride to the sample vials containing the Resin 2 straws, all the oxygen in the sample was consumed, and an uncharacterized black precipitate formed. Several reactions can yield black precipitates with Hg^{2+} (e.g., with H_2S); however, the source of any hydrogen sulfide and the consumption of oxygen remain to be determined.

Section S3. Microbial community composition analysis at the family level

Microbial community composition analysis at the family level revealed detailed differences between the PHA, PP-based, Resin 1, paper straws, and seawater (**Figure S12**). Specifically, seawater was dominated by five families: *Flavobacteriaceae*, *Rhodobacteraceae*, *Methylophilaceae*, *AEGEAN-169 marine group (Alphaproteobacteria)*, and SAR116 clade (*Alphaproteobacteria*). In contrast, the degradable and non-degradable straws had different community compositions, with three major common families: *Microtrichaceae*, *Rhodobacteraceae*, and *Flavobacteriaceae*. In addition to these major common families, the relative abundance of *Arenicellaceae*, *Cycloclasticaceae*, and *Hyphomicrobiaceae* was significantly increased in the three PP-based straws compared with seawater. For the three PHA straws, microbial taxa of *Micavibrionaceae*, *Haliangiaceae*, *Cellvibrionaceae*, and *Burkholderiaceae* were enriched. *Cellvibrionaceae* and *Arenicellaceae* showed significant increases in relative abundance on Resin 1 straws compared with seawater. For the paper straw, *Phycisphaeraceae, Puniceicoccaceae,* and *Cellvibrionaceae* exhibited higher relative abundance than in seawater. Most of the taxa that showed significant changes in relative abundance among the straw samples (e.g., *Cellvibrionaceae*, *Phycisphaeraceae*, and *Puniceicoccaceae*) have been shown to be involved in metabolizing a variety of organic substrates or colonizing plastic surfaces in marine environments.[20,26–29]

Section S4. Satisfying non-sustainability-related design considerations

Each straw worked; they could transfer liquid. However, differences likely exist in consumer preference, a highly subjective measure but arguably one of the most important qualities from a brand and business perspective. For instance, straw material has been shown to correlate with consumer experience when drinking iced tea.[30] Paper straws can get soggy after prolonged use, leading to user dissatisfaction. The Resin 2 straw had a rough texture and was easily fractured when bent, properties that may result in reduced user satisfaction owing to user inertia and familiarity bias. The difference in price between straws was marginal when viewed on an individual straw basis, costing at most 0.08 \$USD/straw. The $CaCO_3$ filled Resin was the least expensive, and the PHA2 and Resin 2 straws were the most expensive. However, drinking straws are sold at extreme volumes (millions of straws), and this price difference can be sizable for businesses (e.g., fast food vendors), potentially contributing to market inertia.

Section S5. Sustainability of seaweed-based materials

Seaweed is considered a sustainable aquaculture crop with many advantages. It sequesters carbon like other biogenic materials and does not require agricultural land, fertilizers, and supplied water in its cultivation, negating many environmental impacts associated with terrestrial crops.[31–34] From a renewability standpoint, seaweed-derived polymers are favorable. Bio-based feedstocks are generally renewable, but their sustainability can vary depending on the agricultural practices used to cultivate the biomass.

Seaweed-derived polymers have the potential to be carbon offsetting. This opportunity is challenged by the intensive processing (e.g., drying and extraction) required to access the polymeric components contained within seaweed, which can counteract the carbon sequestration accomplished by the seaweed biomass. For example, it has been estimated that the embodied GHG emissions to farm and dry sugar kelp (*S. latissima*) can be 6.12 kg CO_2-eq/kg dry mass seaweed.[35] This depends heavily on the energy composition of the processing plant and biorefinery and is expected to decrease with an increase in the amount of electricity derived from non-carbon-emitting sources. In the same analysis, using optimized conditions, the estimated embodied GHG emissions to produce sodium alginate (a biopolymer used in seaweed-based plastics) were modeled to be 0.921 kg CO_2-eq/kg dry mass seaweed.[35] In comparison, using **Equation 1**, the theoretical maximum offset in GHG emissions for *S. latissima* (20-30 wt% C[36,37]) can range from ~0.73 to 1.1 kg CO_2/kg dry mass seaweed. Technological advances in processing seaweed are needed to fully realize the potential reductions in GHG emissions it can afford.

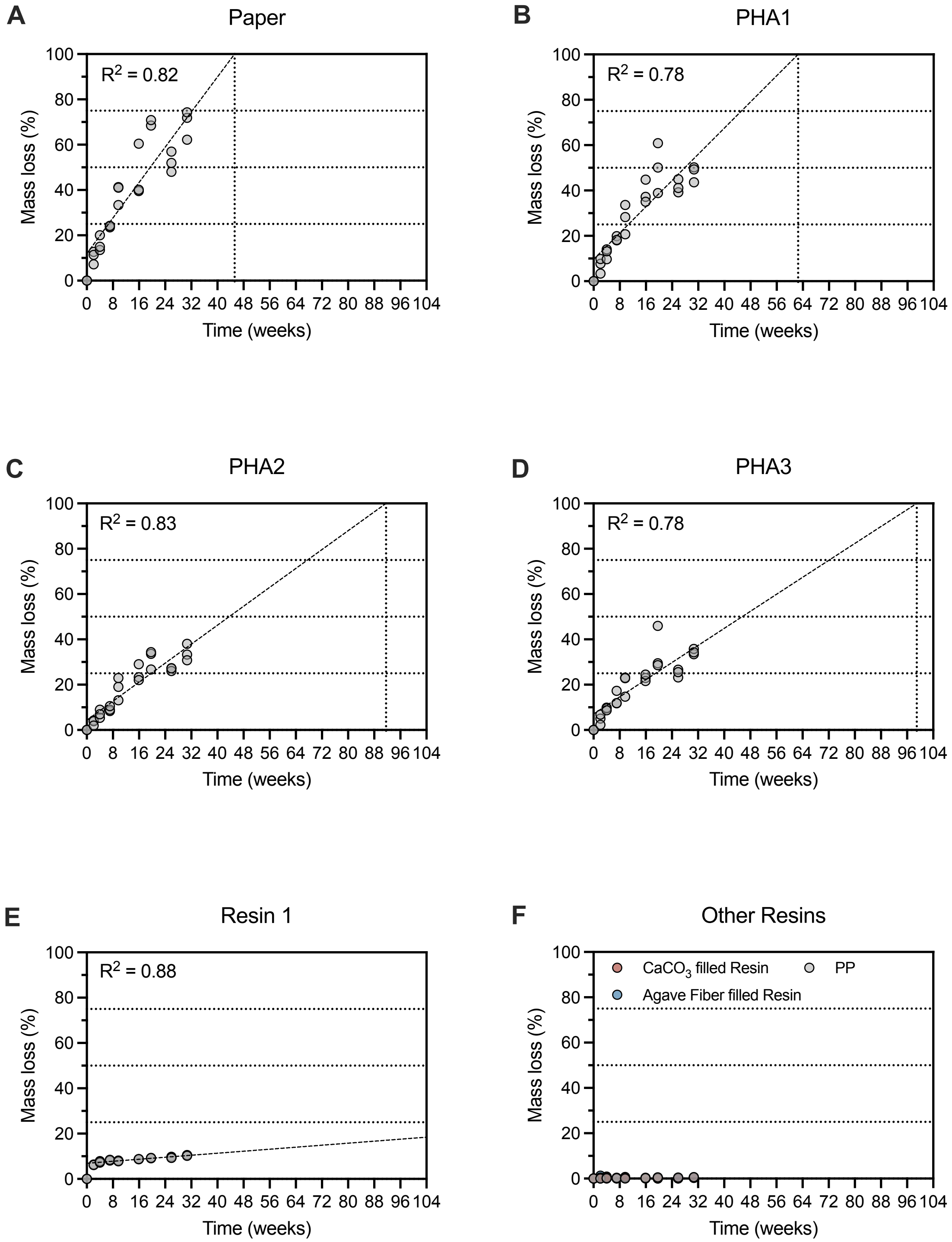

Figure S1. Representative regressions for the (A) paper straw, (B) PHA1 straw, (C) PHA2 straw, (D) PHA3 straw, (E) Resin 1 straw, and (F) the other resin straws. Dashed lines belong to the model fits.

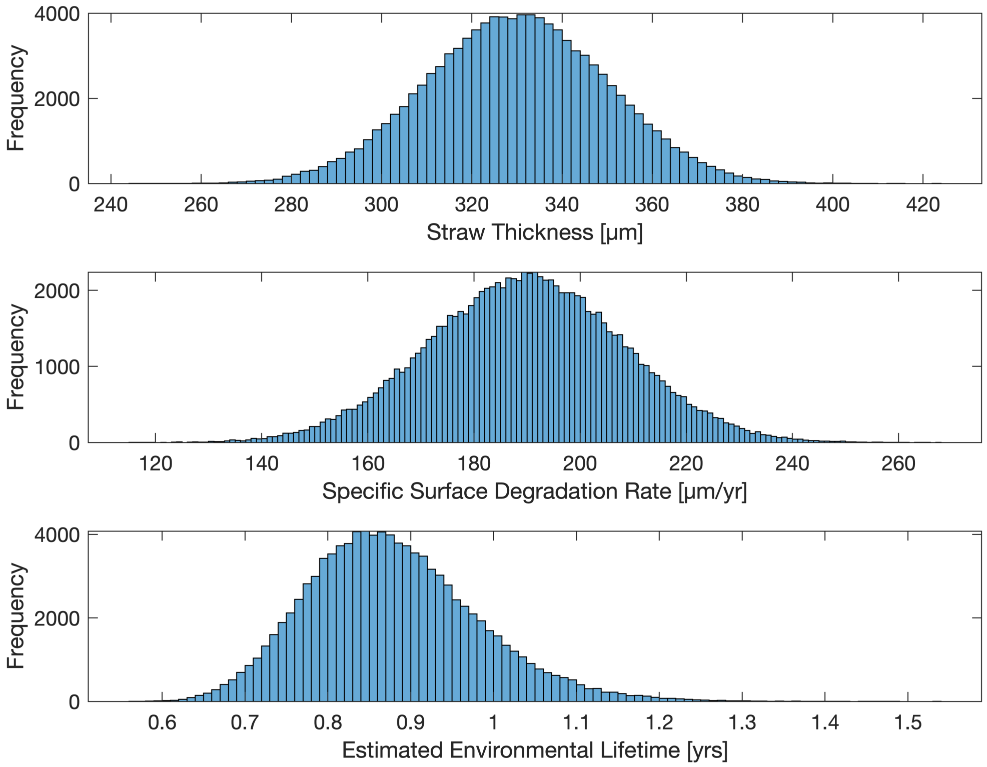

Figure S2. Bootstrapped sampling of straw thickness, specific surface degradation rate (k_d), and estimated environmental lifetime for the paper straw.

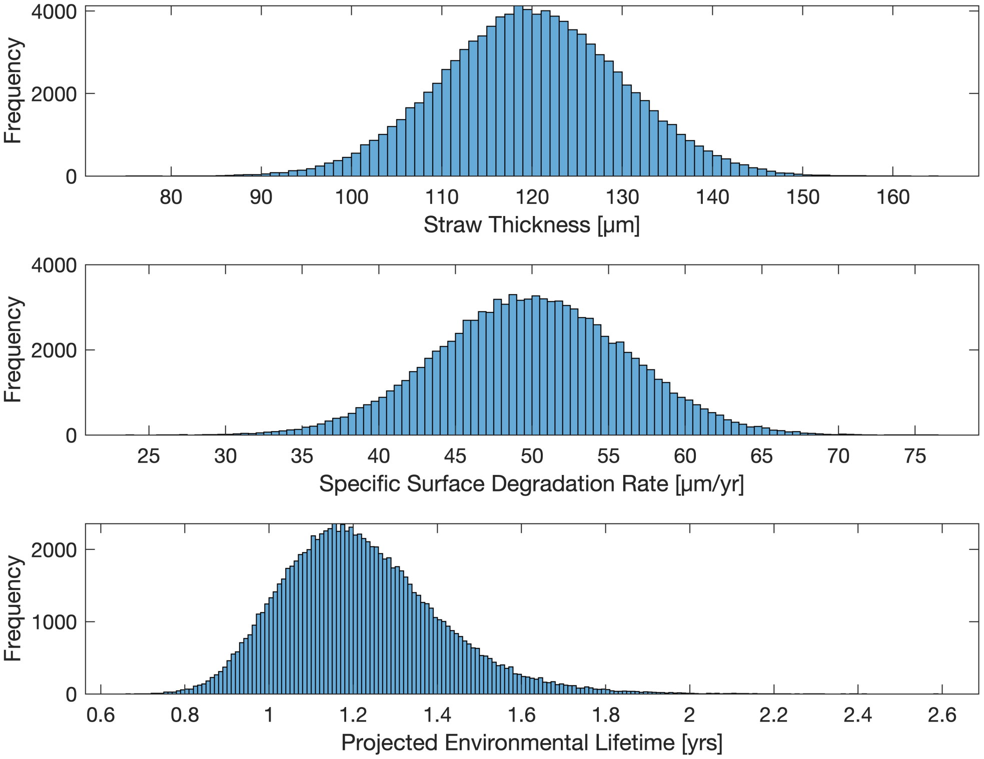

Figure S3. Bootstrapped sampling of straw thickness, specific surface degradation rate (k_d), and estimated environmental lifetime for the PHA1 straw.

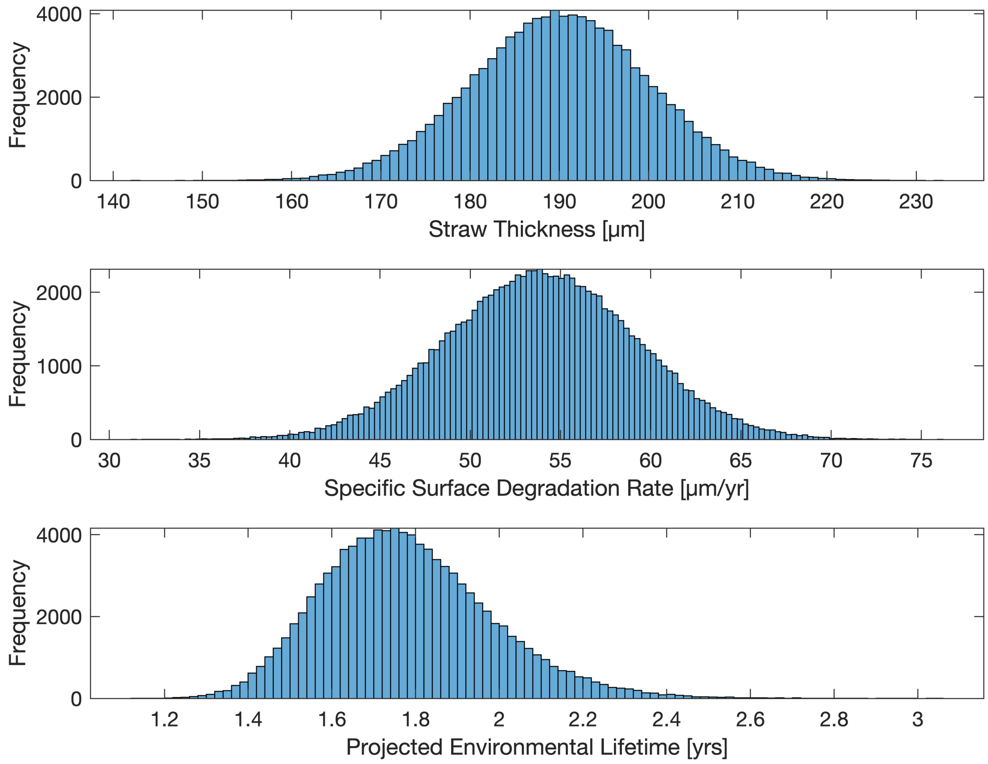

Figure S4. Bootstrapped sampling of straw thickness, specific surface degradation rate (k_d), and estimated environmental lifetime for the PHA2 straw.

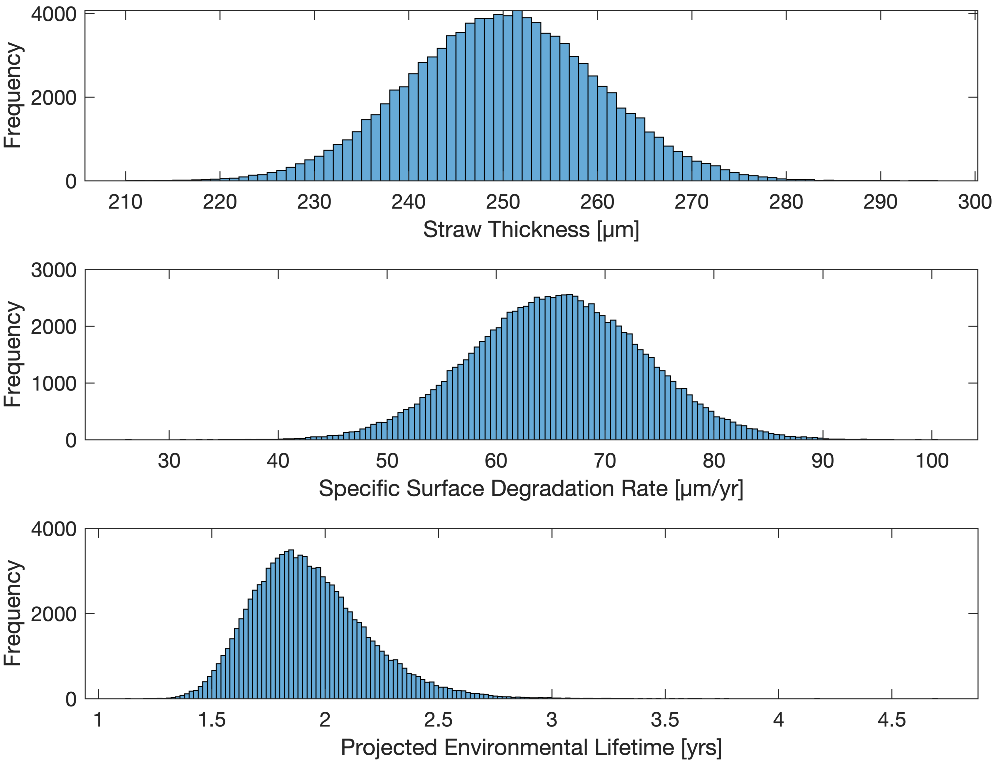

Figure S5. Bootstrapped sampling of straw thickness, specific surface degradation rate (k_d), and estimated environmental lifetime for the PHA3 straw.

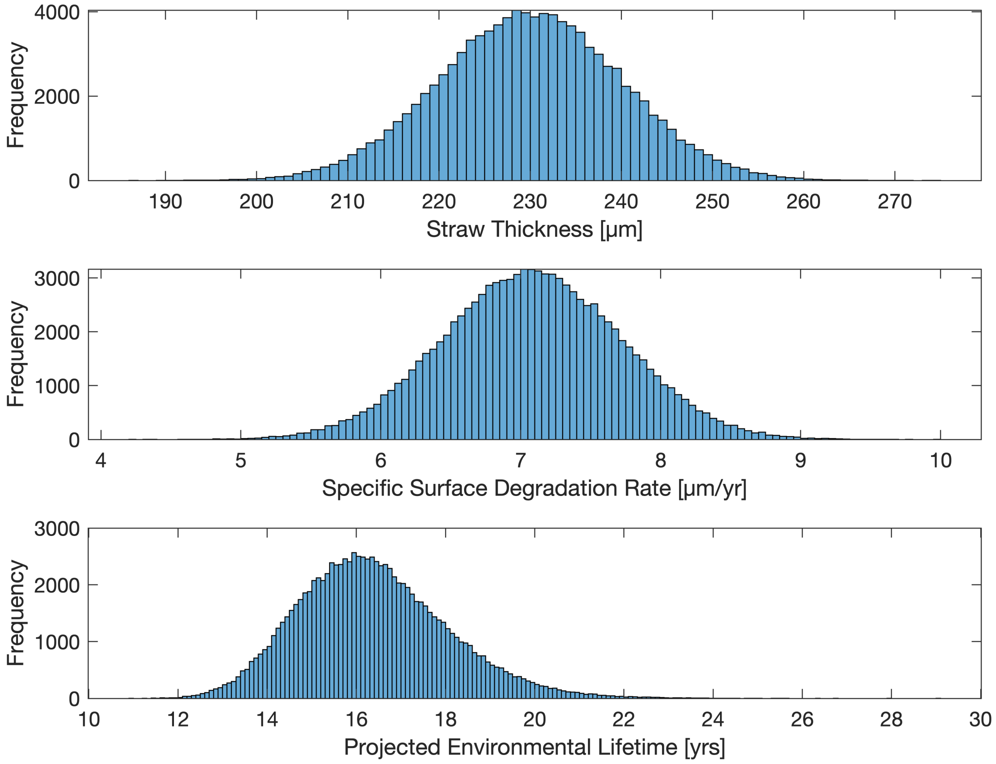

Figure S6. Bootstrapped sampling of straw thickness, specific surface degradation rate (k_d), and estimated environmental lifetime for the Resin 1 straw.

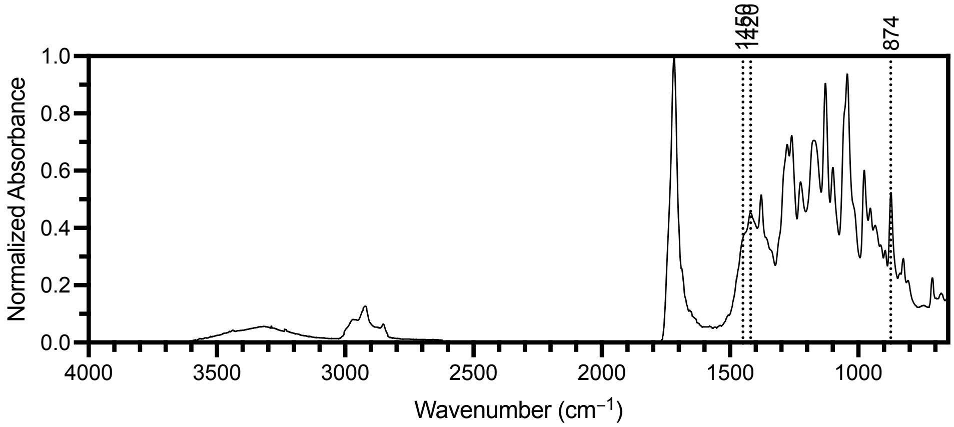

Figure S7. ATR-FTIR spectrum of the PHA2 straw. Characteristic peaks for $CaCO_3$ are labeled.

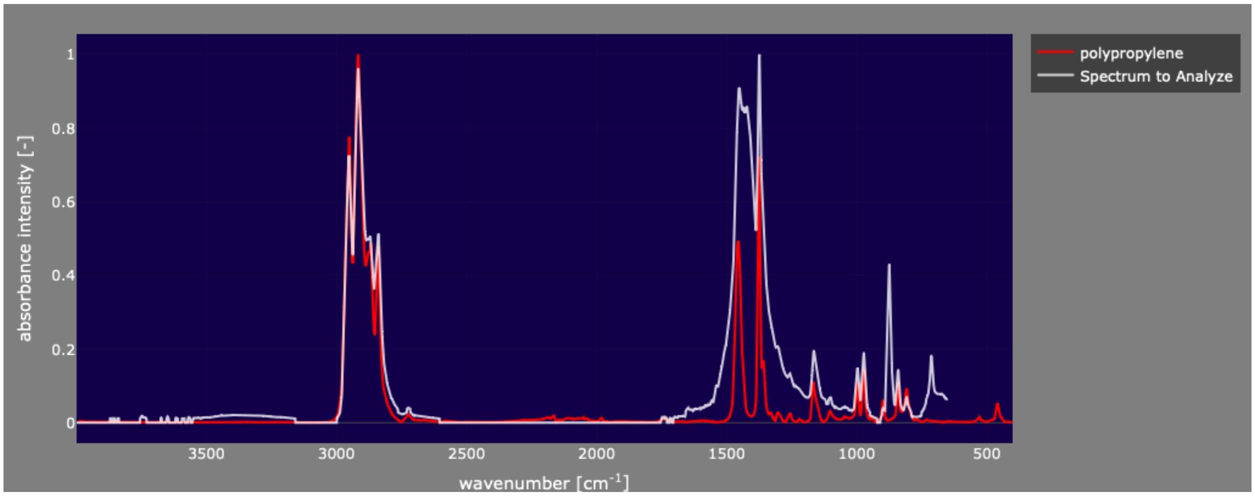

Figure S8. ATR-FTIR spectrum of the $CaCO_3$ filled Resin straw (labeled "Spectrum to Analyze"). The PP reference is in red. The figure was exported from Open Specy.[3] Pearson's r = 0.76.

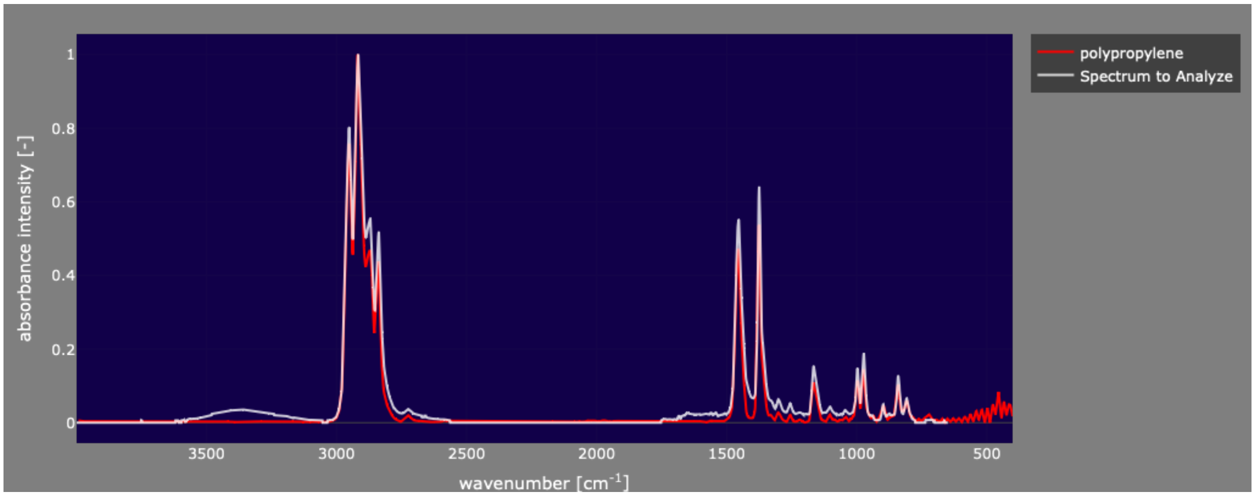

Figure S9. ATR-FTIR spectrum of the Agave Bagasse filled Resin straw (labeled "Spectrum to Analyze"). The PP reference is in red. The figure was exported from Open Specy.[3] Pearson's r = 0.99.

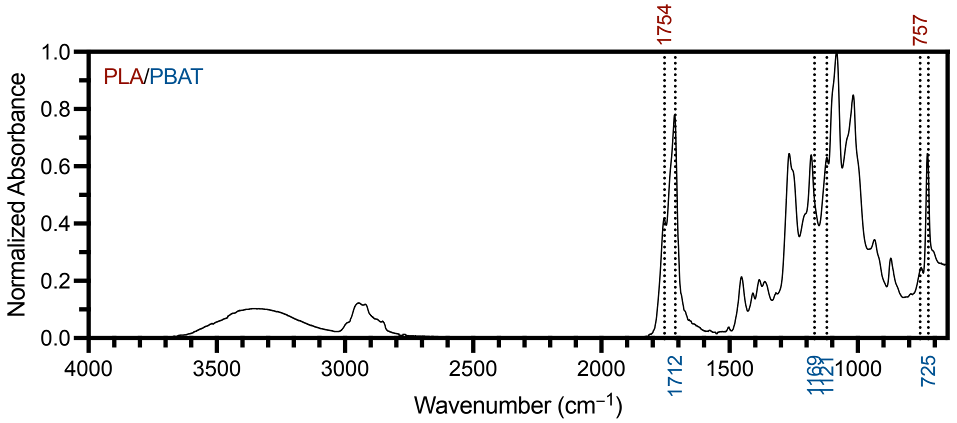

Figure S10. ATR-FTIR spectrum of the Resin 1 straw. Characteristic peaks for PLA and PBAT are annotated in red (above) and blue (below), respectively.

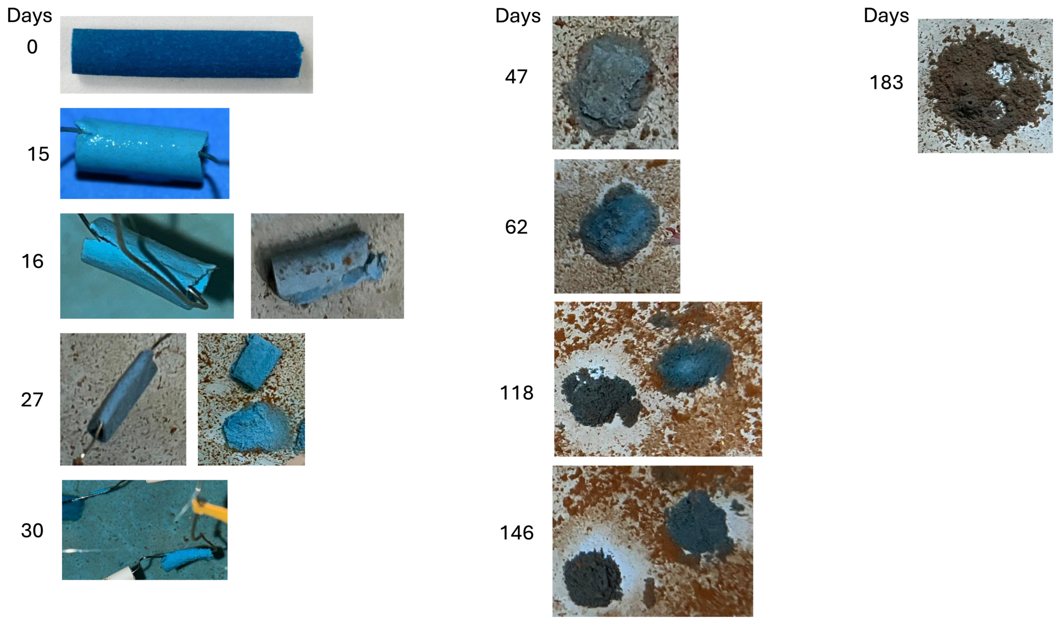

342 **Figure S11.** Time-series of Resin 2 in the mesocosm tank.

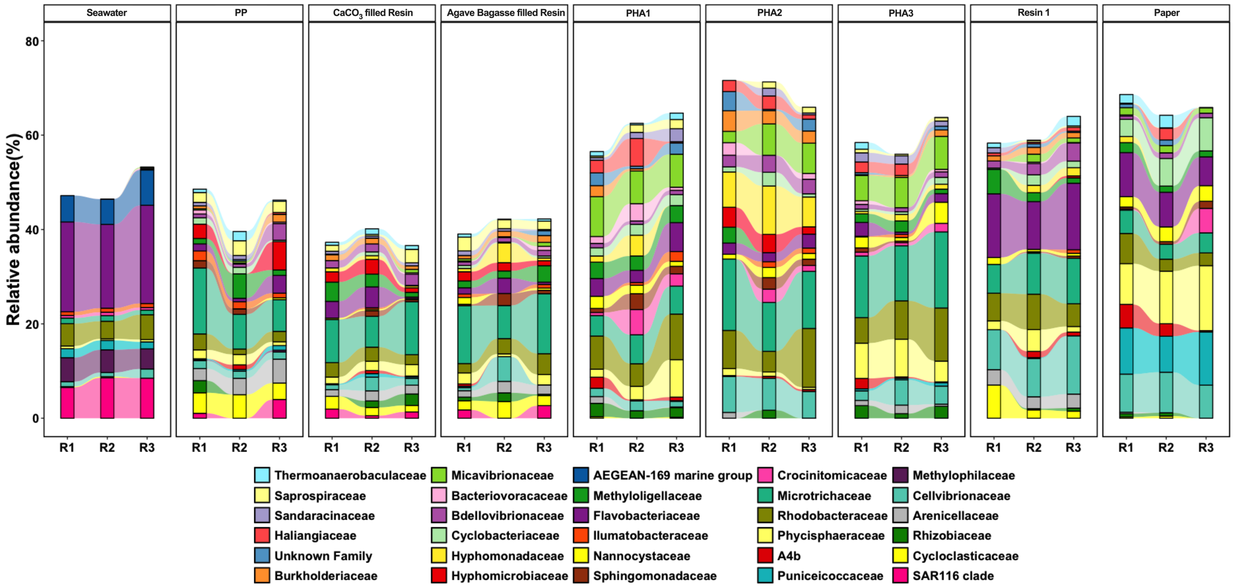

Figure S12. Relative abundance distributions of the top 30 ASVs at the family level growing on the straws and present in the seawater. The numbers shown on the x-axis labels indicate the biological replicate (e.g., replicate 1 as R1).

Table S1. List of some drinking straws on the market

Brand Name	Polymer	URL[a]
Phade	PHA	https://www.phadeproducts.com/#
Beyond Green	PHA	https://byndgrn.com/products/pha-straws
Pura Vida Biolastics	PHA	https://puravidabioplastics.com
Omao	PHA	https://www.omaostraw.com
Beyond Plastic	PHA	https://beyondplastic.com/products/pha-drinking-straw-no-wrap-7-75
D2W	PP	https://ecoqualityinc.com/products/oxo-biodegradable-clear-disposable-jumbo-drinking-ecostraws-7-75in-individually-wrapped-sugarcane-made-d2w-compostable-plastic-alternative?%23+of+Boxes=1
Ecoproducts	PLA	https://ecoproducts.com/7-75in-pla-straw-wrapped-clear-5mm/
Aircarbon	PHA	https://www.aircarbon.com/product/aircarbon-straws
LOLIWARE	Seaweed-derived biopolymer blend	https://www.loliware.com/straw
Matter	Undisclosed biopolymer	https://shop.makeitmatter.com/products/matter-compostable-straws-50-count
Biolo	PHA	https://biolo.com/collections/straws/products/jumbo-straw?variant=42578664423681
Sustainable Agave Company	Undisclosed polymer filled with agave bagasse	https://sustainableagavecompany.com/collections/agave-straws
Strawfish	Undisclosed polymer filled with biogenic calcium carbonate	https://www.strawfish.co
Back to Earth	CDA	https://stonestraw.com/eco-friendly-straws/back-to-earth-straw-2/
Vegware	PLA	https://www.vegwareus.com/us/catalogue/straws/
LifeMade	PHA	https://www.target.com/p/lifemade-earth-friendly-straws-50ct/-/A-82656188
Eagle Beverage Products	CDA and PHA	https://www.eagle-beverage.com/eco-single-use-straw
Urthpact	PHA	https://www.urthpact.com/compostable-straws/
Greenprint	Undisclosed polymer filled with agave bagasse	https://greenprintproducts.com/product/home-agave-straws/

[a]accessed October 29, 2024

Table S2. Drinking straw parameters for select straws investigated by James et al.

Straw	Mass [mg][a]	Thickness [μm][b,c]	k_d [μm/yr][c]	Projected Environmental Lifetime [months][c]
CDA	728.9	170 ± 20	51 ± 7	20 ± 4
PLA	720.2	110 ± 10	< 0.5[d]	> 1320
PHA4	1109	180 ± 10	75 ± 5	15 ± 1
PHA5	916.6	190 ± 10	75 ± 5	15 ± 1

[a]Presented as the mean ± standard deviation of five measurements for the product

[b]Presented as the mean ± standard deviation of seven to ten measurements

[c]Values reported by or recalculated from James et al. out to 16 weeks

[d]Constrained value of k_d determined from geometry and mass loss data; in lieu of biodegradation (enzymatic hydrolysis) in marine settings, PLA is recognized to undergo abiotic hydrolysis, which progresses via bulk degradation and not surface degradation.[38,39] The rate of hydrolysis is highly dependent on temperature (considered to follow an Arrhenius relationship).[38] In the ocean, PLA is expected to persist for decades based on theoretical hydrolysis rates. To date, no long-term study has corroborated these theoretical estimates.[39] Future work is needed to empirically constrain such rates and inform improved estimates of the lifetime of PLA based articles.

Table S3. Mass loss (mg) for each straw during the time series

		Paper			PHA1			PHA2			PHA3		
Collection Date	Time (weeks)	R1	R2	R3	R1	R2	R3	R1	R2	R3	R1	R2	R3
7/5/23	0	0.0	0.0	0.0	0.0	0.0	0.0	0.0	0.0	0.0	0.0	0.0	0.0
7/20/23	2.1	20.1	15.7	10.1	4.9	5.4	1.7	5.1	5.3	2.1	6.0	7.6	2.9
8/2/23	4.0	28.4	20.0	21.1	9.4	5.0	6.9	11.9	6.8	8.6	14.4	10.7	10.9
8/24/23	7.1	36.1	40.2	35.1	8.5	10.0	10.0	10.4	11.6	13.4	12.7	21..0	12.5
9/11/23	9.7	60.7	60.7	47.8	17.2	17.3	13.9	21.1	30.4	17.7	27.2	27.6	21.2
10/24/23	15.9	61.7	61.6	98.6	17.9	19.9	31.8	28.5	28.1	35.4	28.9	22.8	33.0
11/20/23	19.7	112.8	-	114.7	23.7	24.8	32.4	35.9	28.9	44.2	32.1	27.7	48.6
1/2/24	25.9	77.3	67.2	72.7	25.9	24.6	19.9	31.7	28.5	26.4	29.7	33.8	36.1
2/5/24	30.7	97.7	93.3	105.3	25.5	30.8	23.7	46.0	42.4	39.0	38.8	50.2	38.8

		PP			$CaCO_3$ filled Resin			Agave Fiber filled Resin			Resin 1		
Collection Date	Time (weeks)	R1	R2	R3	R1	R2	R3	R1	R2	R3	R1	R2	R3
7/5/23	0	0.0	0.0	0.0	0.0	0.0	0.0	0.0	0.0	0.0	0.0	0.0	0.0
7/20/23	2.1	0.0	0.0	0.0	0.1	0.5	0.3	1.3	0.3	-0.6	-	9.5	7.7
8/2/23	4.0	-0.4	0.0	0.2	0.9	0.2	0.4	0.1	-0.1	0.3	11.9	11.4	11.3
8/24/23	7.1	0.0	0.3	0.2	0.1	-0.2	-0.2	-0.1	-0.1	0.1	11.4	10.1	11.6
9/11/23	9.7	0.3	0.0	-0.3	0.3	0.7	-1.0	-0.1	0.2	0.3	11.8	10.0	10.7
10/24/23	15.9	0.2	0.3	0.2	-0.1	0.2	0.0	-0.1	-0.3	-0.2	11.5	14.1	11.1
11/20/23	19.7	0.3	0.2	0.5	0.0	0.1	0.4	0.0	0.3	0.4	12.1	13.6	13.7
1/2/24	25.9	0.2	0.4	-0.6	-0.4	0.1	-0.4	0.0	0.1	-0.2	15.1	14.2	11.3
2/5/24	30.7	-1.0	0.6	0.5	-0.2	0.4	-0.3	-0.3	0.3	-0.1	13.7	14.9	13.0

Table S4. Relative mass loss (%) for each straw during the time series

		Paper			PHA1			PHA2			PHA3		
Collection Date	Time (weeks)	R1	R2	R3	R1	R2	R3	R1	R2	R3	R1	R2	R3
7/5/23	0	0.0	0.0	0.0	0.0	0.0	0.0	0.0	0.0	0.0	0.0	0.0	0.0
7/20/23	2.1	12.7	11.4	7.2	7.7	9.8	3.4	4.2	3.9	2.0	5.1	6.8	2.3
8/2/23	4.0	20.0	13.6	14.9	14.0	9.7	13.2	9.0	5.4	7.0	9.9	9.5	8.7
8/24/23	7.1	23.6	24.2	24.1	19.9	18.0	18.2	8.4	9.0	10.5	11.8	17.3	11.9
9/11/23	9.7	41.3	41.0	33.4	28.3	33.6	20.7	19.0	22.9	13.1	23.2	22.9	14.7
10/24/23	15.9	39.5	40.2	60.5	37.2	35.0	44.8	23.3	22.1	29.1	22.9	21.6	24.4
11/20/23	19.7	68.5	-	70.9	50.1	38.9	60.9	33.6	26.7	34.3	29.5	28.6	45.9
1/2/24	25.9	57.0	48.0	51.9	45.0	39.2	41.1	27.3	26.1	27.2	23.2	26.6	25.6
2/5/24	30.7	71.8	62.2	74.3	50.2	49.3	43.7	38.1	33.3	30.8	33.5	35.8	34.3

		PP			$CaCO_3$ filled Resin			Agave Fiber filled Resin			Resin 1		
Collection Date	Time (weeks)	R1	R2	R3	R1	R2	R3	R1	R2	R3	R1	R2	R3
7/5/23	0	0.0	0.0	0.0	0.0	0.0	0.0	0.0	0.0	0.0	0.0	0.0	0.0
7/20/23	2.1	0.0	-0.1	0.0	0.1	0.5	0.3	1.3	0.3	-0.6	-	6.4	6.2
8/2/23	4.0	-0.5	0.0	0.2	0.9	0.2	0.4	0.1	-0.1	0.3	7.9	7.2	7.5
8/24/23	7.1	0.0	0.4	0.2	0.1	-0.2	-0.2	-0.1	-0.1	0.1	8.2	8.1	8.5
9/11/23	9.7	0.3	0.0	-0.3	0.3	0.7	-1.0	-0.1	0.2	0.3	8.1	8.0	7.8
10/24/23	15.9	0.2	0.4	0.2	-0.1	0.2	0.0	-0.1	-0.3	-0.2	8.7	8.7	8.8
11/20/23	19.7	0.4	0.2	0.5	0.0	0.1	0.4	0.0	0.4	0.3	9.2	9.1	9.3
1/2/24	25.9	0.2	0.4	-0.7	-0.4	0.1	-0.4	0.0	0.1	-0.2	9.8	9.4	9.3
2/5/24	30.7	-1.1	0.6	0.6	-0.2	0.4	-0.3	-0.3	0.3	-0.1	10.4	10.6	10.2

Table S5. Global warming and resource utilization properties

Straw	Embodied GHG Emissions[a]	Embodied Water Usage[a]	Compost GWP	Landfill GWP
Units	(kg CO_2-eq /kg)	(L/kg)	(kg CO_2-eq/kg)	(kg CO_2-eq/kg)
PHA1	-119.90	240	2.05	12.66
PHA2[b]	1.90[c,d]	202[c,d]	2.09[c]	13.08[c]
PHA3	2.25	240	2.20	14.32
PHA4[b]	2.25	240	2.09[c]	13.08[c]
PHA5[b]	2.25	240	2.09[c]	13.08[c]
Paper	0.71	1700	1.61	9.02
CDA	3.40	240	1.83	10.04
PLA	2.80	270	1.83	10.25
Resin 1	3.73[c]	190[b]	1.87[c]	10.71[c]
PP	2.90	39	0.00	0.00
Agave Bagasse filled Resin	1.80[c]	110[c,e]	2.73[c]	20.80[c]
$CaCO_3$ filled Resin	2.31[c]	32[c]	2.58[c]	20.44[c]

[a]data from references 40–43
[b]assumed 75% 3-hydroxybutyrate and 25% 3-hydroxyhexanoate
[c]calculated by a rule-of-mixtures of the polymer(s) and/or filler/additive
[d]embodied GHG emissions for $CaCO_3$ filler (nonbiogenic), 0.054 CO_2-eq/kg; embodied water usage for $CaCO_3$ filler (nonbiogenic), 0.477 L/kg from reference 45
[e]embodied water usage for producing agave bagasse, 323 L/kg from reference 44

Table S6. Short-term microbial respiration rates

Straw	Respiration Rate [μM O_2/week][a]
Seawater	21.6 ± 0.6
Paper	202.1 ± 7.0
PHA1	87.6 ± 3.9
PHA2	98.6 ± 5.4
PHA3	84.4 ± 6.4
Resin 1	194.0 ± 12.7
$CaCO_3$ filled Resin	29.6 ± 1.2
Agave Bagasse filled Resin	28.3 ± 1.4
PP	37.4 ± 2.5

[a]mean ± standard deviation of three measurements

Table S7. Social costs of GHG emissions and marine plastic pollution

Straw	BoL GHG Emissions (US$/1000 straws)	EoL GHG Landfill (US$/1000 straws)	EoL GHG Compost (US$/1000 straws)	EoL Plastic Pollution (US$/1000 straws)
PHA1	-11.55	0.41	0.20	0.007
PHA2	0.34	0.91	0.37	0.023
PHA3	0.43	0.89	0.42	0.019
PHA4	0.55	1.06	0.51	0.017
PHA5	0.45	0.88	0.42	0.015
CDA	0.55	0.56	0.29	0.015
PLA	0.44	0.56	0.29	0.997
Resin 1	0.76	0.83	0.38	0.213
Paper	0.18	0.77	0.40	0.012
PP	0.39	0.00	0.00	1.152
PP + AB	0.32	1.06	0.49	2.994
PP + $CaCO_3$	0.38	0.88	0.42	2.361

References

(1) Walsh, A. N.; Reddy, C. M.; Niles, S. F.; McKenna, A. M.; Hansel, C. M.; Ward, C. P. Plastic Formulation Is an Emerging Control of Its Photochemical Fate in the Ocean. *Environ Sci Technol* **2021**, *55* (18), 12383–12392. https://doi.org/10.1021/acs.est.1c02272.

(2) Walsh, A. N.; Dunlea, A. G.; Reddy, C. M.; Ward, C. P. Characterization of Inorganic Additives in and Photochemically Liberated from Consumer Plastics: Implications for Global and Local Biogeochemical Cycles. *ACS Environmental Au* **2025**. https://doi.org/10.1021/acsenvironau.4c00130.

(3) Cowger, W.; Steinmetz, Z.; Gray, A.; Munno, K.; Lynch, J.; Hapich, H.; Primpke, S.; De Frond, H.; Rochman, C.; Herodotou, O. Microplastic Spectral Classification Needs an Open Source Community: Open Specy to the Rescue! *Anal Chem* **2021**, *93* (21), 7543–7548. https://doi.org/10.1021/acs.analchem.1c00123.

(4) Walsh, A. N.; Mazzotta, M. G.; Nelson, T. F.; Reddy, C. M.; Ward, C. P. Synergy between Sunlight, Titanium Dioxide, and Microbes Enhances Cellulose Diacetate Degradation in the Ocean. *Environ Sci Technol* **2022**, *56* (19), 13810–13819. https://doi.org/10.1021/acs.est.2c04348.

(5) Mazzotta, M. G.; Reddy, C. M.; Ward, C. P. Rapid Degradation of Cellulose Diacetate by Marine Microbes. *Environ Sci Technol Lett* **2022**, *9* (1), 37–41. https://doi.org/10.1021/acs.estlett.1c00843.

(6) Kana, T. M.; Darkangelo, Christina.; Hunt, M. Duane.; Oldham, J. B.; Bennett, G. E.; Cornwell, J. C. Membrane Inlet Mass Spectrometer for Rapid High-Precision Determination of N2, O2, and Ar in Environmental Water Samples. *Anal Chem* **1994**, *66* (23), 4166–4170. https://doi.org/10.1021/ac00095a009.

(7) Ward, C. P.; Nalven, S. G.; Crump, B. C.; Kling, G. W.; Cory, R. M. Photochemical Alteration of Organic Carbon Draining Permafrost Soils Shifts Microbial Metabolic Pathways and Stimulates Respiration. *Nat Commun* **2017**, *8* (1), 772. https://doi.org/10.1038/s41467-017-00759-2.

(8) Chamas, A.; Moon, H.; Zheng, J.; Qiu, Y.; Tabassum, T.; Jang, J. H.; Abu-Omar, M.; Scott, S. L.; Suh, S. Degradation Rates of Plastics in the Environment. *ACS Sustain Chem Eng* **2020**, *8* (9), 3494–3511. https://doi.org/10.1021/acssuschemeng.9b06635.

(9) Maga, D.; Galafton, C.; Blömer, J.; Thonemann, N.; Özdamar, A.; Bertling, J. Methodology to Address Potential Impacts of Plastic Emissions in Life Cycle Assessment. *Int J Life Cycle Assess* **2022**, *27* (3), 469–491. https://doi.org/10.1007/s11367-022-02040-1.

(10) James, B. D.; Sun, Y.; Pate, K.; Shankar, R.; Izallalen, M.; Mazumder, S.; Perri, S. T.; Houston, K. R.; Edwards, B.; de Wit, J.; Reddy, C. M.; Ward, C. P. Foaming Enables Material-Efficient Bioplastic Products with Minimal Persistence. *ACS Sustain Chem Eng* **2024**, *12* (43), 16030–16040. https://doi.org/10.1021/acssuschemeng.4c05822.

(11) Zhang, L.; Yin, Y.; Sun, Y.; Liang, X.; Graham, D. E.; Pierce, E. M.; Löffler, F. E.; Gu, B. Inhibition of Methylmercury and Methane Formation by Nitrous Oxide in Arctic Tundra Soil Microcosms. *Environ Sci Technol* **2023**, *57* (14), 5655–5665. https://doi.org/10.1021/acs.est.2c09457.

(12) Callahan, B. J.; McMurdie, P. J.; Rosen, M. J.; Han, A. W.; Johnson, A. J. A.; Holmes, S. P. DADA2: High-Resolution Sample Inference from Illumina Amplicon Data. *Nat Methods* **2016**, *13* (7), 581–583. https://doi.org/10.1038/nmeth.3869.

(13) Yilmaz, P.; Parfrey, L. W.; Yarza, P.; Gerken, J.; Pruesse, E.; Quast, C.; Schweer, T.; Peplies, J.; Ludwig, W.; Glöckner, F. O. The SILVA and "All-Species Living Tree Project (LTP)" Taxonomic Frameworks. *Nucleic Acids Res* **2014**, *42* (D1), D643–D648. https://doi.org/10.1093/nar/gkt1209.

(14) Quast, C.; Pruesse, E.; Yilmaz, P.; Gerken, J.; Schweer, T.; Yarza, P.; Peplies, J.; Glöckner, F. O. The SILVA Ribosomal RNA Gene Database Project: Improved Data Processing and Web-Based Tools. *Nucleic Acids Res* **2012**, *41* (D1), D590–D596. https://doi.org/10.1093/nar/gks1219.

(15) Wickham, H. *Ggplot2 Elegant Graphics for Data Analysis*; Springer New York: New York, NY, 2009. https://doi.org/10.1007/978-0-387-98141-3.

(16) McMurdie, P. J.; Holmes, S. Phyloseq: An R Package for Reproducible Interactive Analysis and Graphics of Microbiome Census Data. *PLoS One* **2013**, *8* (4), e61217. https://doi.org/10.1371/journal.pone.0061217.

(17) Oksanen, J.; Blanchet, F. G.; Friendly, M.; Kindt, R.; Legendre, P.; McGlinn, D.; Minchin, P. R.; O'hara, R. B.; Simpson, G. L.; Solymos, P. Vegan: Community Ecology Package. 2019. *R package version* **2015**, *2* (10).

(18) Gebretsadik, T. T.; Tesfay, A. H.; Gebru, A. G.; Assayehegn, E.; Desta, Y. H.; Gebremedhin, K. H.; Gebrehiwet, H.; Teklemedhin, T. B. Characterization and Comparative Insights on *Agave Americana* and *Agave Sisalana* Leaf Fibers for High-Performance Applications. *Journal of Natural Fibers* **2023**, *20* (2). https://doi.org/10.1080/15440478.2023.2246648.
(19) Bengtsson, A.; Bengtsson, J.; Sedin, M.; Sjöholm, E. Carbon Fibers from Lignin-Cellulose Precursors: Effect of Stabilization Conditions. *ACS Sustain Chem Eng* **2019**, *7* (9), 8440–8448. https://doi.org/10.1021/acssuschemeng.9b00108.
(20) James, B. D.; Sun, Y.; Izallalen, M.; Mazumder, S.; Perri, S. T.; Edwards, B.; de Wit, J.; Reddy, C. M.; Ward, C. P. Strategies to Reduce the Environmental Lifetimes of Drinking Straws in the Coastal Ocean. *ACS Sustain Chem Eng* **2024**, *12* (6), 2404–2411. https://doi.org/10.1021/acssuschemeng.3c07391.
(21) BPI. *Search for Certified Compostable Products*. https://products.bpiworld.org (accessed 2024-10-27).
(22) Tüv Austria. *Database of certified products*. https://www.tuv-at.be/okcert/certified-products/ (accessed 2024-10-27).
(23) Stone Straw. *The Compostable Straw*. https://stonestraw.com/eco-friendly-straws/back-to-earth-straw/ (accessed 2025-04-19).
(24) U.S. Food and Drug Administration. *FCN No. 178*. https://www.hfpappexternal.fda.gov/scripts/fdcc/index.cfm?set=FCN&id=178&sort=Sort_FCS&order=DESC&startrow=1&type=advanced&search=¤¤nature%20works¤ (accessed 2025-04-19).
(25) Cargill Dow. *Environmental Assessment*; 2001. https://wayback.archive-it.org/7993/20170606192830/https://www.fda.gov/downloads/Food/IngredientsPackagingLabeling/EnvironmentalDecisions/UCM143101.pdf (accessed 2025-04-19).
(26) Choo, Y.-J.; Lee, K.; Song, J.; Cho, J.-C. Puniceicoccus Vermicola Gen. Nov., Sp. Nov., a Novel Marine Bacterium, and Description of Puniceicoccaceae Fam. Nov., Puniceicoccales Ord. Nov., Opitutaceae Fam. Nov., Opitutales Ord. Nov. and Opitutae Classis Nov. in the Phylum 'Verrucomicrobia.' *Int J Syst Evol Microbiol* **2007**, *57* (3), 532–537. https://doi.org/10.1099/ijs.0.64616-0.
(27) Wynne, E. C.; Pemberton, J. M. Cloning of a Gene Cluster from *Cellvibrio Mixtus* Which Codes for Cellulase, Chitinase, Amylase, and Pectinase. *Appl Environ Microbiol* **1986**, *52* (6), 1362–1367. https://doi.org/10.1128/aem.52.6.1362-1367.1986.
(28) Ortmann, A. C.; Lu, Y. Initial Community and Environment Determine the Response of Bacterial Communities to Dispersant and Oil Contamination. *Mar Pollut Bull* **2015**, *90* (1–2), 106–114. https://doi.org/10.1016/j.marpolbul.2014.11.013.
(29) Wright, R. J.; Langille, M. G. I.; Walker, T. R. Food or Just a Free Ride? A Meta-Analysis Reveals the Global Diversity of the Plastisphere. *ISME J* **2021**, *15* (3), 789–806. https://doi.org/10.1038/s41396-020-00814-9.
(30) Pramudya, R. C.; Singh, A.; Seo, H.-S. A Sip of Joy: Straw Materials Can Influence Emotional Responses to, and Sensory Attributes of Cold Tea. *Food Qual Prefer* **2021**, *88*, 104090. https://doi.org/10.1016/j.foodqual.2020.104090.
(31) Zhang, X.; Boderskov, T.; Bruhn, A.; Thomsen, M. Blue Growth and Bioextraction Potentials of Danish Saccharina Latissima Aquaculture — A Model of Eco-Industrial Production Systems Mitigating Marine Eutrophication and Climate Change. *Algal Res* **2022**, *64*, 102686. https://doi.org/10.1016/j.algal.2022.102686.
(32) Froehlich, H. E.; Afflerbach, J. C.; Frazier, M.; Halpern, B. S. Blue Growth Potential to Mitigate Climate Change through Seaweed Offsetting. *Current Biology* **2019**, *29* (18), 3087-3093.e3. https://doi.org/10.1016/j.cub.2019.07.041.
(33) Laurens, L. M. L.; Lane, M.; Nelson, R. S. Sustainable Seaweed Biotechnology Solutions for Carbon Capture, Composition, and Deconstruction. *Trends Biotechnol* **2020**, *38* (11), 1232–1244. https://doi.org/10.1016/j.tibtech.2020.03.015.
(34) Duarte, C. M.; Bruhn, A.; Krause-Jensen, D. A Seaweed Aquaculture Imperative to Meet Global Sustainability Targets. *Nat Sustain* **2021**, *5* (3), 185–193. https://doi.org/10.1038/s41893-021-00773-9.
(35) Nilsson, A. E.; Bergman, K.; Gomez Barrio, L. P.; Cabral, E. M.; Tiwari, B. K. Life Cycle Assessment of a Seaweed-Based Biorefinery Concept for Production of Food, Materials, and Energy. *Algal Res* **2022**, *65*, 102725. https://doi.org/10.1016/j.algal.2022.102725.

(36) Gevaert, F.; Davoult, D.; Creach, A.; Kling, R.; Janquin, M.-A.; Seuront, L.; Lemoine, Y. Carbon and Nitrogen Content of *Laminaria Saccharina* in the Eastern English Channel: Biometrics and Seasonal Variations. *Journal of the Marine Biological Association of the United Kingdom* **2001**, *81* (5), 727–734. https://doi.org/10.1017/S0025315401004532.

(37) Umanzor, S.; Stephens, T. Nitrogen and Carbon Removal Capacity by Farmed Kelp Alaria Marginata and Saccharina Latissima Varies by Species. *Aquaculture Journal* **2022**, *3* (1), 1–6. https://doi.org/10.3390/aquacj3010001.

(38) Laycock, B.; Nikolić, M.; Colwell, J. M.; Gauthier, E.; Halley, P.; Bottle, S.; George, G. Lifetime Prediction of Biodegradable Polymers. *Prog Polym Sci* **2017**, *71*, 144–189. https://doi.org/10.1016/j.progpolymsci.2017.02.004.

(39) Read, T. Degradation of Biodegradable Plastics in Aquatic Environments, University of Queensland, 2025. https://doi.org/10.14264/f2db5d4.

(40) Ashby, M. F. *Materials and the Environment*, 2nd ed.; Elsevier, 2013. https://doi.org/10.1016/C2010-0-66554-0.

(41) Aircarbon. *Aircarbon Straws*. https://www.aircarbon.com/product/aircarbon-straws (accessed 2024-10-27).

(42) Lee, S.; Lee, I.; Seo, D.; Kim, H.; Joo, G.; Lee, S.; Park, K. Life Cycle Assessment of APHA Production. *ACS Sustain Chem Eng* **2024**, *12* (1), 72–84. https://doi.org/10.1021/acssuschemeng.3c04788.

(43) Luo, C.; Zhou, Y.; Chen, Z.; Bian, X.; Chen, N.; Li, J.; Wu, Y.; Yang, Z. Comparative Life Cycle Assessment of PBAT from Fossil-Based and Second-Generation Generation Bio-Based Feedstocks. *Science of The Total Environment* **2024**, *954*, 176421. https://doi.org/10.1016/j.scitotenv.2024.176421.

(44) Davis, S. C.; Kuzmick, E. R.; Niechayev, N.; Hunsaker, D. J. Productivity and Water Use Efficiency of *Agave Americana* in the First Field Trial as Bioenergy Feedstock on Arid Lands. *GCB Bioenergy* **2017**, *9* (2), 314–325. https://doi.org/10.1111/gcbb.12324.

(45) Calcium Carbonate Association – Europe. *Dry ground calcium carbonate (GCC-Dry) Ultrafine - Sector EPD*. https://api.environdec.com/api/v1/EPDLibrary/Files/de625bc2-b69f-434f-6990-08d9b3ca1199/Data (accessed 2025-08-19).
